# A Multi-Model Based Stability Analysis Employing Multi-Environmental Trials (METs) Data for Discerning Heat Tolerance in Chickpea (*Cicer arietinum* L.) Landraces

**DOI:** 10.3390/plants12213691

**Published:** 2023-10-26

**Authors:** Thippeswamy Danakumara, Tapan Kumar, Neeraj Kumar, Basavanagouda Siddanagouda Patil, Chellapilla Bharadwaj, Umashankar Patel, Nilesh Joshi, Shayla Bindra, Shailesh Tripathi, Rajeev Kumar Varshney, Sushil Kumar Chaturvedi

**Affiliations:** 1 ICAR—Indian Agricultural Research Institute, New Delhi 110012, India; danakumarat@gmail.com (T.D.); neeraj0490@gmail.com (N.K.); bs_patil2000@yahoo.com (B.S.P.); umesh.gpb94@gmail.com (U.P.); niluameta99@gmail.com (N.J.); 2 International Centre for Agricultural Research in the Dry Areas, Sehore 466113, Madhya Pradesh, India; tapanbio@gmail.com; 3 Department of Plant Breeding & Genetics, Punjab Agricultural University, Ludhiana 141027, Panjab, India; shaylabindra@pau.edu; 4 ICAR—Indian Institute of Pulses Research, Kanpur 282004, Uttar Pradesh, India; shaitri@rediffmail.com; 5 Centre for Crop & Food Innovation, Murdoch University, Murdoch, WA 6150, Australia; rajeev.varshney@murdoch.edu.au; 6 Department of Genetics & Plant Breeding, Rani Lakshmi Bai Central Agricultural University, Jhansi 284003, Uttar Pradesh, India; sushilk.chaturvedi@gmail.com

**Keywords:** AMMI, WAASB, GGE, chickpea, heat susceptibility index

## Abstract

Identifying a congenially targeted production environment and understanding the effects of genotype by environmental interactions on the adaption of chickpea genotypes is essential for achieving an optimal yield stability. Different models like additive main effect and multiplicative interactions (AMMI 1, AMM2), weighted average absolute scores of BLUPs (WAASB), and genotype plus genotype–environment (GGE) interactions were used to understand their suitability in the precise estimation of variance and their interaction. Our experiment used genotypes that represent the West Asia–North Africa (WANA) region. This trial involved two different sowing dates, two distinct seasons, and three different locations, resulting in a total of 12 environments. Genotype IG 5871(G1) showed a lower heat susceptibility index (HSI) across environments under study. The first four interactions principal component axis (IPCA) explain 93.2% of variations with significant genotype–environment interactions. Considering the AMMI stability value (ASV), the genotypes IG5862(G7), IG5861(G6), ILC239(G40), IG6002(G26), and ILC1932(G39), showing ASV scores of 1.66, 1.80, 2.20, 2.60, and 2.84, respectively, were ranked as the most stable and are comparable to the weighted average absolute scores of BLUPs (WAASB) ranking of genotypes. The which–won–where pattern of genotype plus genotype–environment (GGE) interactions suggested that the target environment consists of one mega environment. IG5866(G10), IG5865(G9), IG5884(G14), and IG5862(G7) displayed higher stability, as they were nearer to the origin. The genotypes that exhibited a superior performance in the tested environments can serve as ideal parental lines for heat-stress tolerance breeding programs. The weighted average absolute scores of BLUPs (WAASB) serve as an ideal tool to discern the variations and identify the stable genotype among all methods.

## 1. Introduction

Chickpea (*Cicer arietinum* L.) is an important grain legume crop with a high protein content, implying its significance in sustaining food and nutritional security for inhabitants of arid and semiarid regions [1,2,3]. Chickpea-growing regions are predominantly located in arid and semiarid areas, making them susceptible to high-temperature stress, mainly during the reproductive and grain-filling stages [4,5,6,7,8]. The impact of heat stress on chickpea yield and production has become a matter of significant concern in recent times. The paradigm shift in chickpea cultivation is driving factors including a shift in major cropping areas from cooler long-season climates to shorter warm-season climates, the expansion of late-sown chickpea areas due to higher cropping intensities, and also the overall rise in seasonal temperatures caused by global climate change [9,10,11]. From germination to the vegetative and reproductive phases, all stages of chickpea growth are hindered by temperatures exceeding the normal range [12,13,14,15,16,17]. During the terminal stage of chickpea growth, which encompasses the reproductive phase, heat stress can have significant consequences for both crop yield and quality. This critical stage involves the formation of flowers and pods and the development of seeds [18,19,20].

There is an urgent need to identify stable sources of donors that can be utilized into a breeding program [21,22]. The identification of landraces known for their acclimatization under harsh and hardy environments is a basic prerequisite. Testing their response to Indian agroclimatic conditions for normal and late-sown conditions can provide crucial clues about genotypes with inherent heat-stress-tolerant capacities [23,24]. Genotype–environment interaction (GEI) occurs when different genotypes show varying responses to changing environments with fluctuations in crop yield. Researchers rely on stable statistical measures that include both univariate and multivariate techniques. These methods encompass cluster analysis, pattern analysis, and principal component analysis (PCA) or biplots. Biplots, in particular, are highly effective graphical tools used to represent the relationships between genotypes and environments visually to analyze genotype–environment interactions (GEIs). Two widely utilized biplot models are the AMMI biplot (additive main effects and multiplicative interaction) and the GGE biplot (genotype-plus-genotype × environment) [25,26,27,28,29]. Researchers commonly employ genotype-plus-genotype by environment (GGE) biplots for various purposes [30,31,32], such as classifying mega environments, assessing genotype rankings, and selecting discriminative and representative environments. Best linear unbiased prediction (BLUP) had a higher predictive accuracy than any AMMI family member in analyzing multi-environment trials (METs) with a random analysis of the genotype–environment interaction (GEI) effect using a linear mixed-effect model (LMM). The weighted average absolute scores of BLUPs (WAASB) can reliably estimate stability in a bidimensional plot with multiple interactions on the principal component axes (IPCAs), which is essential for ranking genotypes. The simultaneous selection index WAASBY is useful when different weights are needed for performance and stability [33,34,35,36]. 

In this study, we used multi model analysis to identify high-yielding, stable chickpea genotypes across diverse environments using various stability analyses.

## 2. Results

According to Fischer and Maurer (1978) [37], the HSI (heat susceptibility index) has served as the predominant criterion for identifying tolerant genotypes. In our study, HSI was calculated using timely and late-sown data of three locations for two consecutive years. Results showed that IG 5871 (0.12, 0.12, 0.18, 0.07, 0.1, 0.08) had a lower HSI across environments and across locations, along with ILC 8666, IG 5905, and IG 5868, which exhibited lower yield dips under more than four environments (Supplementary Table S1).

### 2.1. Variance Analysis for Yields

The combined analysis of variance (Table 1) revealed highly significant differences (*p* ≤ 0.001, *p* ≤ 0.01) for yield per plot among different factors: locations, genotypes, and the genotype–location (G × E) interaction.

**Figure 1:**
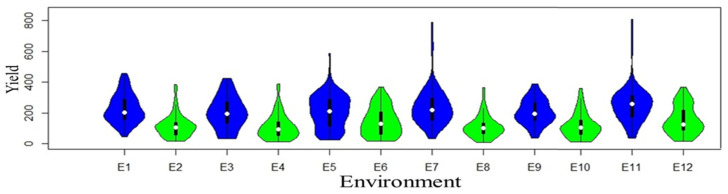
Violin plots of yield trait of each individual environment are distributed for both normal (blue color) and late (green color) conditions.

Yield variations result from evolving environments and genetic diversity. Each violin plot (Figure 1) consists of two mirrored distributions, one for normal-sown conditions (blue color) and the other (green color) for late-sown conditions.

The width of each violin corresponds to the density of data points, with broader sections indicating higher data density and narrower sections indicating lower density. The plots illuminate differences in central tendencies, spread, and distribution shapes, offering valuable insights into the impact of late conditions on crop yields. Genotype performance variation was prominent among locations, with 18.47% attributed to location, 4.03% to replication, 16.19% to genotype, and the highest, 21.30%, to genotype–environment interaction across locations. Moreover, qualitative (crossover type) interaction was demonstrated where the rank order of genotypes changed across environments (Supplementary Figure S1A and Table S2), indicating the presence of substantial genotype–environment interactions. The overall average yield recorded 174.5 g/plot, with the minimum value of 110 g/plot in the 2021 Dharwad late-sown condition, and the maximum yield value of 257 g/plot recorded in the 2022 Delhi timely-sown condition (Supplementary Figure S1B). Annichiarico environmental index was used to predict favorable and unfavorable environments along with the most suitable genotype for the particular environment (Supplementary Table S3). Descriptive statistics, variance components, and genetic parameters for grain yield of all the genotypes evaluated across the year and locations are provided in Supplementary Table S4, where overall heritability (broad sense) across environments was reported to be 72.54%. To analyze genotype–environment interaction (GEI), a principal component analysis (PCA) was executed (Table 1). The ordination method using an approximate F-statistic showcased significant distinctions across PC1 (35.4%), PC2 (28.9%), and PC3 (21.7%), totaling 86% variation. Inclusion of PC4 (7.2%) led to a cumulative 93.2% explanation of variability, with a significant (*p* < 0.001) effect.

### 2.2. AMMI Biplot Analyses

#### 2.2.1. Additive Main Effects and Multiplicative Interaction: AMMI 1

**Figure 2:**
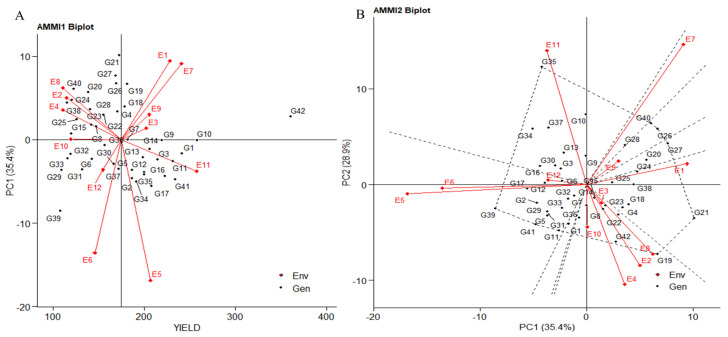
(**A**) The “AMMI 1” biplot depicts the primary effects of traits, and the first principal component (PC1) impacts of both genotype and environment. (**B**) The “AMMI 2” biplot visualizes the combined effects of the first two principal components (PC1 and PC2) for genotypes along with the genotype-environment interaction impact of 42 genotypes across two seasons and three locations, considering yield per plot (g/plot).

The biplot displays the first principal component (PC1) term on the abscissa and significant trait influence on the ordinate. Environments E3, E9, and E10, representing plot yield, showed PCA1 scores closer to zero, parallel to the average yield line. This suggests the strong performance of all genotypes in these environments. E6, E5, and E1 had vectors parallel to PC1 (35.4%), indicating a higher contribution to overall variation. E4, E2, E8, E1, E7, E9, and E3 had higher average yields than E10, aligned with the average yield line. E12, E6, E5, and E11 were below-average environments (Figure 2A). Certain genotypes like IG5862(G7) and ILC1312(G36) consistently excelled across all environments, with high mean yields near the axis origin. Genotypes ILC1312(G36), IG5856(G4), IG5904(G18), and IG5980(G21) also had higher mean yields, while IG5858(G5) and ILC1313(G37) had lower yields, aligning with zero scores on the first PCA1 axis. These genotypes were less affected by environmental variations. 

#### 2.2.2. Additive Main Effects and Multiplicative Interaction: AMMI 2

The extent of interaction is reflected by the distance of the environment and genotype vectors from the biplot’s origin. Shorter vectors closer to the origin suggest less interaction, serving as a reliable indicator for selecting genotypes with consistent performance and adaptability. For example, E6, E9, E7, E5, and E1 align parallel to PC1 (35.4%). Notably, E5 and E6 are closely related with a smaller angle in the negative quadrant, while E1, E9, and E7 are correlated in the positive quadrant. Dashed lines in the biplot highlight genotype contributions to specific environments. Genotypes IG6000(G24), IG6001(G25), ILC184 (G38), IG5904(G18), IG5856(G4), and IG5980(G21) are associated with E1, while IG6002(G26), IG5909(G20), and ILC239(G40) exhibit shorter vectors, contributing to E9. In the positive quadrant of PC1, E7 correlates with genotype IG6006(G28). Conversely, in the negative quadrant of PC1, E6 and E5 connect with genotypes ILC1932(G39), ILC8666(G41), IG5868(G11), G2, ILC0(Austria)(G29), IG5896(G17), IG5874(G12), IG5861(G6), ILC0(Greece)(G31), and IG5858(G5). E1’s vectors demonstrate negative interactions compared to E5 and E6. PC2 variation (28.9%) attributes influence to E10, E11, E4, E2, and E6, aligning parallel to the PC2 axis (Figure 2B). Notably, genotypes IG5866(G10), IG5865(G9), and IG5862(G7) exhibit superior performance, particularly in E11. IG5862(G7) aligns with the negative quadrant of PC2 alongside E10. E11 negatively correlates with E3, E8, E2, E4, and E10, while E3, E10, E4, E2, and E8 have positive relationships contributing to PC2. Genotypes clustered near the biplot origin, such as IG5862(G7), IG5863(G8), IG5997(G23), ILC1312(G36), ILC0(Italy)(G32), IG5886(G15), and IG5884(G14), demonstrate consistent responses across environments. Conversely, genotypes positioned far apart, like ILC10771(G35), IG5905(G19), and ILC1932(G39), exhibit distinct environmental responses. Genotypes located farther from the origin display heightened sensitivity to environmental interactions. The angle between environment and genotype vectors signifies the correlation coefficient and interaction degree. A right angle implies no correlation (E1 to E4, E11 to E6), while obtuse and acute angles suggest negative (E11 to E10, E4, E2, E8) and positive correlations (E3, E9, E7, and among E2, E4, E8, E10), respectively. In the PC1 vs. PC3 biplot (Supplementary Figure S2A), genotypes IG5862(G7), ILC1312(G36), IG5866(G10), and IG5863(G8) cluster near the origin. Shortest vectors contributing to PC1 belong to E2, E9, and E8. Meanwhile, E10, with the shortest vector, contributes to PC3, demonstrating more variation against E3, with involvement of genotypes IG5866(G10), IG5884(G14), and IG5865(G9). In the PC2 vs. PC3 biplot, genotypes IG5896(G17), ILC0(Italy)(G32), IG5862(G7), and IG5874(G12) cluster near the origin (Supplementary Figure S2B). Environments E6 and E1 showcase stability with the shortest vector length.

### 2.3. WAASBY-Based Genotype Ranking for Stability and High Performance

**Figure 3:**
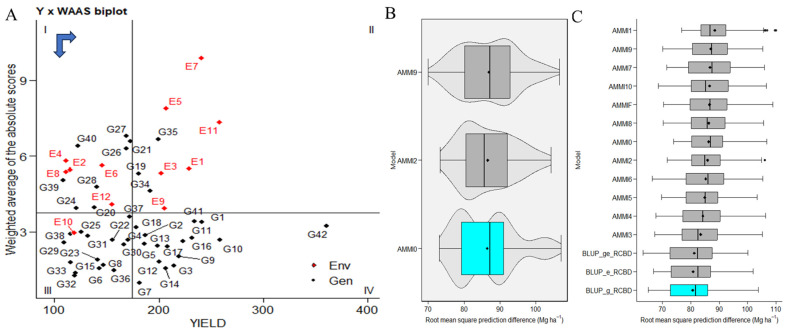
(**A**) The biplot presents the relationship between grain yield and the weighted average of absolute scores for the best linear unbiased prediction of genotype–environment interaction (WAASB) for 42 genotypes tested across 12 environments. A black circle in the IV quadrant represents a theoretical genotype with both high productivity and broad adaptability. Horizontal and vertical black arrows indicate the directions of yield increase and stability improvement, respectively. (**B**,**C**) The comparative predictive precision of the AMMI (additive main effects and multiplicative interaction) family and best linear unbiased prediction (BLUP) methods for the yield of 42 *C. arietinum* genotypes are depicted. The distribution of 1000 estimates of root mean square prediction difference (RMSPD) is visualized through boxplots.

WAASBY values used fixed weights of 65% for grain yield (GY) and 35% for stability (WAASB). The highest WAASBY values belong to genotypes ILC239(G40), IG6002(G26), IG5862(G7), ILC0(Czech)(G30), IG5861(G6), ILC1932(G39), IG5997(G23), ILC8666(G41), IG5856(G4), G5895(G16), IG6003(G27), JG14(G42), IG5865(G9), IG6000(G24), IG5858(G5), ILC184(G38), IG5993(G22), IG5863(G8), and ILC0(Russia)(G34). These genotypes are positioned in quadrant IV, indicating both high productivity and stability (Figure 3A). In contrast, changing genotype rankings based on assigned weights are visualized in Supplementary Figure S3A. The left side presents ranks based solely on stability, while moving from left to right and from top down increases the weight for productivity by 5% in each scenario. The right-most side corresponds to genotype ranking for grain yield. Cluster analysis in this study tries to identify IG5856(G4) and groups with similar stability and productivity performance. In Cluster 1, genotypes ILC1312(G36), ILC10771(G35), ILC0(Latvia)(G33), ILC0(Greece)(G31), ILC0(Austria)(G29), IG6006(G28), IG5980(G21), IG5909(G20), IG5905(G19), and IG5884(G14) exhibit poor productivity and instability, consistently ranking low in all WAASB/GY ratios. In Cluster 2, genotypes ILC239(G40), ILC1932(G39), ILC184(G38), ILC1313(G37), ILC0(Italy)(G32), IG6001(G25), IG6000(G24), IG5997(G23), IG5993(G22), IG5904(G18), IG5886(G15), IG5863(G8), and IG5861(G6) demonstrate productivity but instability, ranking well with low WAASB/GY ratios. Cluster 3 includes genotypes JG14(G42), ILC8666(G41), ILC0(Czech)(G30), IG6003(G27), IG6002(G26), IG5895(G16), IG5865(G9), IG5862(G7), and IG5856(G4), which exhibit high productivity and broad adaptability, indicating greater stability with lower WAASB values. In Cluster 4, genotypes ILC0(Russia)(G34), IG5896(G17), IG5878(G13), IG5874(G12), IG5868(G11), IG5866(G10), IG5858(G5), IG5852(G3), and IG5842(G2) are stable but low in productivity, ranking well with high WAASB/GY ratios (Supplementary Figure S3B).

### 2.4. Comparison of BLUP and AMMI Family Models

The evaluation determines the superior model in a given context. Based on our diverse datasets exhibiting various genotype–environment interaction (GEI) patterns, our analysis suggested that best linear unbiased prediction (BLUP) emerged as the most predictively precise model (Figure 3B). Furthermore, we observed that AMMI0 demonstrated the highest accuracy within the AMMI models. Among the best linear unbiased prediction (BLUP) models of environment, genotypes, and genotypes by environment, in conjunction with AMMI (0–10 and AMMIF), our observations point to the best linear unbiased prediction (BLUP) by genotypes as the most accurate model.

### 2.5. GEI Biplot Considering WAASB and ASV

**Figure 4:**
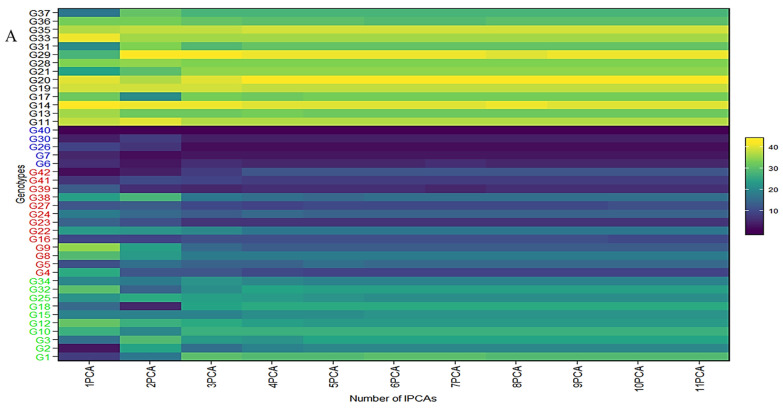

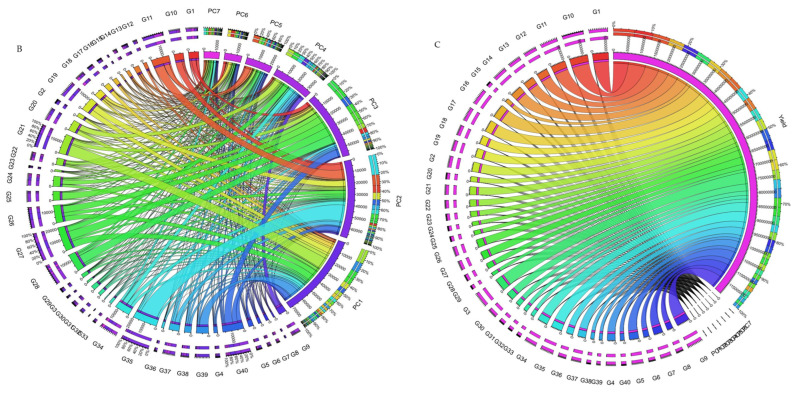
(**A**) The heatmap illustrates the rankings of 42 chickpea genotypes based on the utilization of interaction principal component axes (IPCAs) in the weighted average of absolute scores for the best linear unbiased predictions (BLUPs) of genotype–environment interaction (WAASB) estimation. (**B**) Circos plot showing genotypes contribution to individual IPCAs. (**C**) Circos plot of individual genotypes contribution to yield and respective IPCAs.

The collective variance captured by the initial three interaction principal component analyses (IPCAs) in the chickpea trial amounted to 86% (Table 1). Figure 4 offers a lucid representation of the “which–won–where” pattern. An illustrative example involves JG14(G42) emerging as the prevailing performer across all studied environments in the first IPCA. Its supremacy is attributed to the highest yield (360.86 g/plot) and the lowest IPCA1 score (2.76) among the tested genotypes (Supplementary Table S6). Notably, a correlation exists between Figure 4 and Figure S3. For instance, genotypes IG5896(G17), ILC8666(IG5874(G12)), IG5895(ILC8666(G41)), and IG5874(IG5895(G16)) showcase the most elevated predicted means (207.9, 233.7, 228.7, 200.1) (Figure 4), consequently earning the distinction of “universal winners” due to their minimal IPCA1 scores (−5.59, −4.77, −4.36, and −3.9) (Supplementary Table S6). When examining genotype ranking based on the number of retained interaction principal component axes (Figure 4A–C), eleven interaction principal component axes (IPCAs) were considered for the chickpea dataset (Supplementary Table S6). It is apparent that the genotype ranking was significantly impacted by the number of interaction principal component axes (IPCAs) employed in weighted average absolute scores of BLUPs (WAASB) estimation, particularly up to four interaction principal component axes (IPCAs). Clear groups of genotypes with akin stability performance are discernible by the color variations on the left side. For instance, genotypes ILC239(G40), IG6002(G26), IG5862(G7), ILC0(Czech)(G30), and IG5861(G6) showcase the lowest WAASB values (0.77, 0.93, 1.03, 1.10, and 1.21) when considering four or more interaction principal component axes (IPCAs) (Supplementary Table S6). They are consecutively ranked as the first, second, third, fourth, and fifth most stable. Parallel rankings are observed with the WAAS index for these genotypes (0.98, 1.27, 1.35, 1.46, and 1.54) in the first cluster (dark blue gradient) as we observed a correlation between ASV and weighted average absolute scores of BLUPs (WAASB) (Supplementary Figure S4). However, when accounting for ASV, genotypes IG5862(G7), IG5861(G6), ILC239(G40), IG6002(G26), and ILC1932(G39) receive ASV scores of 1.66, 1.80, 2.20, 2.60, and 2.84, respectively. We observed significant correlation among stability models (Supplementary Figure S4). Consequently, ILC1932(G39) would be ranked as the fifth most stable (Supplementary Figure S3), although in actuality, it belongs to the second cluster of genotypes (red color) with the highest stability.

### 2.6. GGE Biplot Based Analysis

#### 2.6.1. “Which–Won–Where” Pattern

**Figure 5:**
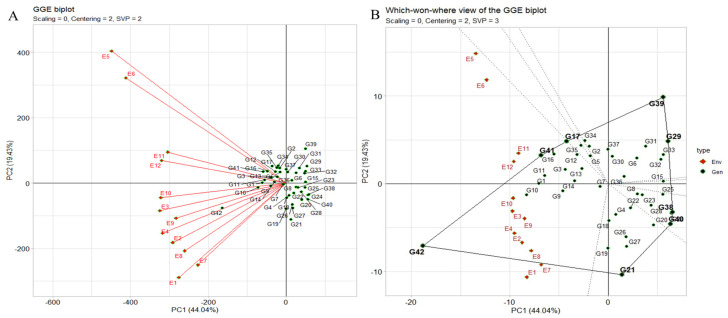
(**A**) The environment vector perspective of the GGE biplot illustrates the resemblances among test environments. (**B**) The GGE biplot polygon view depicting the “which-won-where” pattern showcases the combined impact of genotype main effects and G × E interaction for 42 *C. arietinum* genotypes across two seasons and three locations in terms of yield per plot. These biplots were generated using centering = 2, SVP = 3, and scaling = 0 settings.

The “which–won–where” pattern of multi-environment trials (METs) vividly illustrates genotype–environment interaction (GEI) by correlating genotypes and environments. It also aids in evaluating genotypes stability versus mean performance across environments and assessing test environments for representativeness and discrimination (Figure 5A). The polygon view depicting the “which–won–where” pattern showcases the combined impact of genotype main effects, with the genotype ILC8666(G41) and IG5896(G17) positioned along the line connecting JG14(G42) and ILC1932(G39) affirmed the ranking order JG14(G42) > ILC8666(G41) > IG5896(G17) > ILC1932(G39) across all environments. These equality lines partitioned the biplot into sectors, with the leading genotype for each sector located at the corresponding vertex. In this instance, the twelve environments were grouped into one sector. JG14(G42) emerged as the universal winner, particularly in E9 and E10, while ILC1932(G39) exhibited subpar performance in these environments displaying the crossover (Figure 5A,B).

#### 2.6.2. The Assessment of Stability across Environments Using the “Mean vs. Stability” Analysis of the GGE Biplot

**Figure 6:**
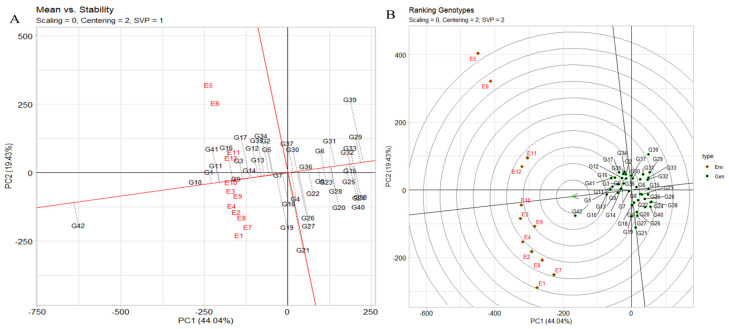
(**A**) The GGE biplot’s depiction of the “mean vs. stability” pattern. (**B**) The GGE biplot “genotypes ranking” pattern for genotype comparison with ideal genotype showing G + G × E interaction effect of 42 genotypes under two seasons and three locations for yield per plot. The biplots were created based on centering = 2, SVP = 2, and scaling = 0.

The assessment of stability across environments using the “mean vs. stability” analysis of the genotype plus genotype by environment (GGE) biplot is an effective approach. In Figure 6A, the abscissa of the average-environment coordination (AEC) indicates a higher mean yield across various environments. Genotypes such as JG14 (G42), IG5871(G1), and IG5868(G11) exhibit the highest mean yields, while closely trailing are IG5895(G16), IG5865(G9), IG5852(G3), and others. IG5980(G21) demonstrates a mean yield similar to the grand mean, in contrast to ILC1932(G39), which exhibits the lowest mean yield. The AEC ordinate, represented by the vertical line, signifies greater variability (indicative of poorer stability) in either direction. Notably, IG5980(G21) and ILC1932(G39) are characterized by high instability, while IG5866(G10), IG5865(G9), IG5884(G14), and IG5862(G7) showcase considerable stability. The instability of ILC1932(G39) stems from its unexpectedly lower yield in several environments (E1, E7, E8, E2, E4, E9, E3, and E10) juxtaposed with higher-than-anticipated yield in E12, E11, E6, and E5.

#### 2.6.3. Genotype Ranking

The genotype ranking biplot, as depicted in Figure 6B, serves as a valuable tool for discerning superior-performing genotypes in comparison to others. Notably, genotypes such as JG14(G42), IG5871(G1), IG5868(G11), IG5866(G10), IG5865(G9), IG5884(G14), IG5858(G5), IG5895(G16), ILC8666(G41), and IG5874(G12) stand out as top contenders due to their close proximity to the arrowhead within the circle. The ranking of genotypes based on yield per plot follows the order JG14(G42) > IG5866(G10) > IG5871(G1) > IG5868(G11) > IG5865(G9) > IG5884(G14) > IG5874(G12) > IG5895(G16) > IG5878(G13) > IG5858(G5) > ILC8666(G41) > IG5896(G17) > ILC10771(G35). Environmental rankings, on the other hand, exhibit the sequence E9 > E10 > E9 > E12 > E11 > E4 > E2 > E8 > E7 > E1. The distance between genotypes on the biplot corresponds to the Euclidean distance, reflecting disparities in mean yield (g) and/or interactions with environments. Longer vectors denote superior (e.g., IG5895(G16)) or inferior (e.g., ILC0 (Austria)(G29), ILC239(G40)) performance, as well as heightened instability (e.g., ILC1932(G39)). Acute angles (e.g., IG5866(G10) vs. IG5865(G9)) imply similar responses with proportional variations across all environments, while obtuse angles (e.g., JG14(G42) vs. ILC1932(G39)) indicate inverse responses, signifying instances where one genotype excels while another falters. Right angles signify independent reactions to environments (e.g., IG5866(G10) vs. ILC1313(G37)), underscoring differences that predominantly influence GE. Comparison between any two genotypes can be contrasted using a connecting line (Supplementary Figure S5A). Performance superiority is towards the side of the equality line. For instance, JG14(G42) excelled in E9, E10, E4, E2, E8, E7, and E1, while ILC1932(G39) outperformed in the remaining environments (E12, E11, E6, and E5), revealing a “crossover” interaction. Variation in genotype disparity across environments correlates with the environment’s distance from the equality line. Differences between JG14(G42) and ILC1932(G39) were notable in E1 and E5 but minor in E9.

#### 2.6.4. The GGE Biplot’s “Discriminativeness vs. Representativeness”

**Figure 7:**
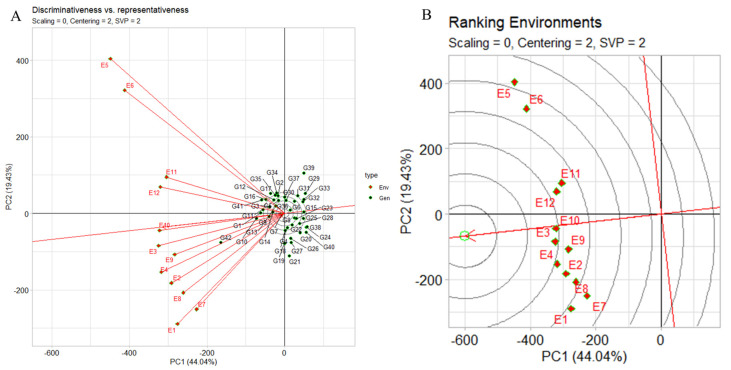
(**A**) GGE biplot “discriminativeness vs. representativeness” pattern compares genotypes, highlighting the ideal genotype with G + G × E interaction. (**B**) GGE biplot “Env. Ranking” pattern compares environments, highlighting ideal environment with G + G × E interaction for 42 genotypes, 2 seasons, and 3 locations, using yield per plot. Biplots: centering = 2, SVP = 2, and scaling = 0.

The genotype plus genotype by environment (GGE) biplot’s “discriminativeness vs. representativeness” pattern serves as a crucial tool for discerning the most suitable test environments to select superior genotypes. The pattern discriminates among genotypes (discriminativeness), and, secondly, faithfully represents the overall set of evaluated environments (representativeness). In Figure 7A, our focus was on yield evaluations across distinct research locations (E9, E8, E2, E11, and E7). The concise vectors associated with these environments offer a clear insight into their discriminative potential. Environments characterized by expansive vectors, such as E5 and E6, exert a stronger discriminatory influence due to their larger variability. For instance, as depicted in the case of environment two (E10, E3, E12, E9) for yield, a subtle angle alongside an extended vector indicates an environment’s heightened representativeness and discriminativeness. Moving to Figure 7A, environment rankings distinctly identify E10 and E3 as prime candidates for yield-focused genotype selection. Conversely, E5, E6, E7, and E1 are relegated to weaker positions, suggesting limited suitability for this purpose. In our evaluation of the three assessed locations, Dharwad emerges as the optimal choice, followed by Amlah and then Delhi. Our analysis of the angles between the AEC abscissa and the test environment vector further accentuates the differentiation between favorable and less ideal environments. Smaller angles, exemplified by environments like E10, E3, E9, E4, E12, E11, and E2, signify more advantageous conditions for genotype selection. Conversely, wider angles, observed in environments such as E5, E6, E1, E8, and E7, imply reduced potential for accurate genotype discrimination. Finally, the length of each environment vector provides a visual representation of its discriminatory capacity. Longer vectors, such as those associated with E5, E6, and E1, are more effective at distinguishing genotypes, emphasizing their significance in the selection process. Conversely, shorter vectors like E3, E10, and E12 display a diminished discriminatory ability.

#### 2.6.5. Test Environment Discrimination and Representation

Figure 7B illustrates distances among test environments for all traits. Concentric circles in the biplot visualize environment vector lengths, proportional to standard deviations within environments, reflecting environment discrimination capability. E5 and E6 are most informative, whereas E10 and E9 are least discriminative. Representativeness of test environments: E10 is highly representative, unlike E5 and E6 which are less so. Environments that are both discriminative and representative, like E10, suit broad genotype selection. Nonrepresentative yet discriminative test environments (e.g., E5, E6) aid specific megaenvironment genotype selection. From Figure 7B rankings, preferred target environments are E10 > E3 > E4 > E12 > E9 > E11 > E2 > E8 > E1 > E7 > E6 > E5. When focusing on a single mega environment, discriminative but nonrepresentative test environments (e.g., E5) effectively eliminate unstable genotypes. With regard to optimal environments for selecting broadly adapted genotypes, in a singular mega environment context, E10 emerges as the premier choice due to its proximity, while E5 and E6 prove suboptimal for region-wide cultivar selection. Notably, E5 and E6, sourced from Delhi_2021 timely- and late-sown seasons, contrast with E10 from Dharwad_2022 late-sown environment. Interrelationships among test environments (Supplementary Figure S5B) portray connections between test environments. E10, E3, E9, E2, E8, and E1 exhibit positive correlation (acute angle), unlike E5 and E1, with no apparent correlation (right angle). The distance between environments quantifies their distinctiveness in genotype differentiation. Nine environments cluster into two groups: E12, E11, E6, and E5 in one, and the remaining in another. Covariance, influenced by vector length and angle cosine, defines similarity between environments. Genotype assessment and ranking (Supplementary Figure S6A) utilizes genotype plus genotype by environment (GGE) biplots to evaluate genotype performance in specific settings. Genotype and environment vectors are depicted, with a 90° angle signifying above-average genotype performance in a specific environment. IG5874(G12) underperforms (obtuse angles) across all environments, while IG5863(G8) excels (acute angles) in all but E7. Genotype ranking for E10 places JG14(G42), IG5866(G10), IG5871(G1), IG586(G11), and IG5865(G9) sequentially. Notably, IG5866(G10) and IG5865(G9) maintain yields close to the average, differing from others yielding higher. JG14(G42) shines in E5, whereas ILC239(G40) ranks lowest. Environment performance evaluation (Supplementary Figure S6B) assesses test environments based on IG5871(G1) performance. A line through the biplot origin and genotype marks the axis, depicting environment rankings. IG5871(G1) yields results below average in E10, E3, and E9, near average in E10 and E12, and above average in remaining environments. 

## 3. Discussion

The integration of analysis of variance and principal component analysis (PCA) has yielded valuable insights into the substantial impacts of location, genotype, and their interactions on yield per plot. Our study underscores the pivotal role of genotype–environment interactions in achieving optimal yields across diverse conditions. The first three PCs explain 86% of the variability, surpassing the minimum reliability threshold of 70% [38,39]. In our comprehensive analysis, which accounts for four PCs, the explanatory power extends to 93.2%, demonstrating significant reliability [40,41,42]. The AMMI 1 analysis, depicted in the results, provides intricate insights into genotype–environment interactions. This approach facilitates the identification of genotypes excelling in specific environments and those showcasing consistent performance across diverse conditions. Notably, ILC1312(G36) and IG5862(G7) emerge as appealing choices for breeders due to their combined high yield and stability [43,44,45]. AMMI 2, a graphical representation using PC1 and PC2 scores, holds distinct advantages over joint-regression-based analysis. It offers a concise overview of complex genotype–environment interactions (GEIs) across multiple settings, elucidating 86% of the G + G × E interaction variation for yield per plot. Thus, the interaction of the 42 genotypes across 12 environments is encapsulated by these three principal components of genotype and environment [46,47]. By studying various scenarios, researchers can evaluate how genotype rankings shift with varying weights assigned to productivity and stability. This approach yields valuable insights for genotype selection in breeding programs. To predict yield, a judicious selection between best linear unbiased prediction (BLUP) and additive main effects and multiplicative interaction (AMMI) models is crucial; our findings align with those of Piepho (1994) [48,49,50,51], who concluded that best linear unbiased prediction (BLUP) surpasses any member of the additive main effects and multiplicative interaction (AMMI) family in yield prediction within the context of multi-environment trials (METs) considering intrinsic factors specific to each trial [52,53,54,55]. The “which–won–where” pattern suggests diverse cultivar selection according to mega environments. The biplot origin represents an average genotype across environments, with proximity indicating minor G and GE contributions. Longer vectors signify higher G or GE contributions. Noteworthy genotypes with extended vectors (e.g., JG14(G42), IG5866(G10)) exhibit superior performance, while others (e.g., IG5980(G21), ILC1932(G39)) show lower stability [56,57]. The angle between genotype vectors and the AEA reflects G and GE components. Certain environments, like E9, E10, and E3, possess notable representativeness and discrimination [58]. In this study for genotype selection, we observed associations between test environments, suggesting potential genotype information reduction, leading to cost-effective testing [59]. Correlations between closely correlated test environments over time enable the omission of one without significant data loss [60]. Our genotype ranking biplot offers critical insights into performance variations across diverse environments, considering mean yield and interactions. This approach holds potential for substantial genotype information reduction, ultimately reducing testing expenses [61,62].

## 4. Materials and Methods

### 4.1. Plant Materials

Forty-one landraces representing the WANA (West Asia and North Africa) region were obtained from the Chickpea Molecular Biology Laboratory, Division of Genetics, IARI, New Delhi (Supplementary Table S7), with one variety, JG14, used as a heat-tolerant check. We cumulatively sowed 160 landraces on the same locations and seasons for heat-tolerance screening (data not presented) and stability analysis, but for better representations of biplots we studied 42 genotypes by using the heat susceptibility index (HSI). The chosen 42 profile genotypes were determined by evaluating grain yield per plot while taking into account the heat susceptibility index (HSI) among all the tested landraces.

### 4.2. Environments and Intercultural Practices

Phenotypic evaluation spanned three locations: ICARDA—Amlaha (23.14711° N, 76.92035° E, MSL 502 m) with medium black and alluvial soil, IARI-Regional Research Station—Dharwad (15.45890° N, 75.00780° E, MSL 678 m), with tropical black clays derived from the weathering of metavolcanic rocks as block cotton soil, and IARI—New Delhi (28.08° N, 77.12° E, MSL 228.61 m), with very deep, somewhat excessively drained alluvial soils. Locations with genotypic code along with latitude, longitude, altitude, year as seasons, date of sowing, and date of harvesting are given in the Supplementary Table S7, with two sowing dates, two seasons (30 days apart), and three locations (2 × 2 × 3) cumulatively representing 12 environments, as coded by E1 to E12. A total of 41 landraces with JG214 check underwent field trials over 2021–2022, including normal and late sowing (30 days apart), and temperature and precipitation of each location and season are given in Supplementary Table S8. An alpha lattice design was used, with three replications per location. Randomized lines/checks occupied 2 m plots. Plants were spaced at 30 cm, rows at 50 cm, plots at 1.5 m, and replications at 2.0 m. Standard agronomic practices were diligently adhered to at each respective location. The background effect of drought was minimized by giving an adequate amount of water by artificial irrigation at all the critical stages of the chickpea crop plant.

### 4.3. Data Collection and Statistical Analysis

The seed yield was recorded after weighing the seeds from a two-meter row plot for each genotype; mean yield with their standard deviations are given in Supplementary Table S9. This study aimed to assess the heat susceptibility index (HSI) and categorize genotypes based on their tolerance to heat stress [37], using heat susceptibility index calculated by (S): (1 − Yh/Yn)/(1 − Xh/Xn), where Yh: yield of genotype under heat stress, Yn: yield of genotype under normal condition, stress intensity = 1 − Xh/Xn, Xh: average yield of all genotypes under heat stress, and Xn: average yield of all genotypes under normal condition. The high-heat-tolerant (HSI < 0.50), heat-tolerant (HSI: 0.51–0.75), moderately heat-tolerant (HSI: 0.76–1.00), and heat-susceptible (HSI > 1.00) scores of all the genotypes were estimated (Fischer and Maurer 1978) using Microsoft Excel. Quantitative traits underwent ANOVA to assess variations among genotypes, locations, seasons, and genotype–environment interactions (genotype by location, genotype by season, and genotype by location by season).

The Annicchiarico method, developed in 1992 [63], relies on the recommendation index (ωi(g)), which measures both stability and genotypic adaptability. 

Multivariate stability and G × E interaction analysis was performed by using genotype plus genotype by environment (GGE) biplots and AMMI in R Studio (a simplified version of R). Metan facilitated genotype plus genotype by environment (GGE) biplots, while metan managed AMMI analysis [64,65,66], highlighting biplots, and percent of GE interaction was computed from total sum of squares. Genotype plus genotype by environment (GGE) biplots and AMMI graphically showcased G × E interaction and genotype ranking based on mean and stability. Broad-sense heritability was estimated (H^2^ = VG/VP) for plot yield using BLUP values across the locations and seasons by dividing genotypic variation to total variation [67]. When dealing with high-dimensional data in biplot analysis, both the AMMI and GGE approaches rely on principal component analysis (PCA). However, as the number of components required to capture a significant portion of the original variance increases, it can become challenging to visually interpret the results. In such situations, researchers may need to create multiple biplots to effectively understand the underlying variability in the original genotype–environment interaction (GEI) data (https://power.larc.nasa.gov/data-access-viewer/ (accessed on 10 October 2023)).

## 5. Conclusions

Unveiling genotype adaptability and stability using multi-environment trials (METs) of chickpea genotypes provided crucial insights into their adaptability and stability under varying environmental conditions. Notably, the significance of both genotype and interaction effects was remarkably pronounced (*p* < 0.001). In particular, the evaluation of genotypes ILC239(G40), IG6002(G26), IG5862(G7), ILC0(Czech)(G30), and IG5861(G6) unveiled their remarkable adaptability, with the lowest weighted average absolute scores of BLUP (WAASB) values (0.77, 0.93, 1.03, 1.10, 1.21) observed when considering four or more interaction principal component axes (IPCAs). These genotypes demonstrated resilience across a spectrum of scenarios, as affirmed by the multifaceted statistical results derived from genotype plus genotype by environment (GGE) and AMMI biplots. Comparatively, best linear unbiased prediction (BLUP) outperformed any member of the AMMI family in accuracy. The multi-environment trials (METs) approach unraveled the intricate dynamics of chickpea genotypes, illuminating their versatility and robustness. This study lays a solid foundation for informed genotype selection and breeding advancements, aiming towards bolstered chickpea yields and resilience in the face of varying environmental challenges.

## Figures and Tables

**Table 1:** Estimation of significant level for yield and yield contributed traits of 42 *C. arietinum* accessions revealed by ANOVA.

Source of Variation	Df	Sum Sq	Mean Sq	F Value	Pr (>F)	Proportion	Accumulated
ENV	11	4,159,096	378,099.6 ***	9.99863	1.66 × 10^−6^		
REP(ENV)	24	907,563.5	37,815.15 ***	8.84942	1.25 × 10^−28^		
GEN	41	3,644,107	88,880.67 ***	20.79967	1.24 × 10^−105^		
GEN: ENV	451	4,795,863	10,633.84 ***	2.488509	1.5634 × 10^−32^		
PC1	51	1,699,822	33,329.84 ***	7.8	0	35.4	35.4
PC2	49	1,383,687	28,238.5 ***	6.61	0	28.9	64.3
PC3	47	1,041,098	22,151.02 ***	5.18	0	21.7	86
PC4	45	344,720.9	7660.464 ***	1.79	0.0013	7.2	93.2
PC5	43	158,258.7	3680.435	0.86	0.726	3.3	96.5
PC6	41	62,198.48	1517.036	0.36	0.9999	1.3	97.8
PC7	39	49,545.98	1270.41	0.3	1	1	98.8
PC8	37	22,607.39	611.0104	0.14	1	0.5	99.3
PC9	35	16,737.69	478.2197	0.11	1	0.3	99.6
PC10	33	9285.812	281.3882	0.07	1	0.2	99.8
PC11	31	7901.49	254.8868	0.06	1	0.2	100
Residuals	984	4,204,807	4273.178				
Total	1962	22,507,299	11,471.61				
Overall Mean	174.5151						
CV	37.45783						

Df, degree of freedom; * significant at *p* ≤ 0.05; ** significant at *p* ≤ 0.01; *** highly significant at *p* ≤ 0.001; ns = nonsignificant, *p* > 0.05.

## Data Availability

Data are contained within the article or Supplementary Materials.

## Supplementary Materials

The following supporting information can be downloaded at: https://www.mdpi.com/article/10.3390/plants12213691/s1, Figure S1: Plots showing the pictorial representation of genotype and overall mean performance of studied traits at each environment under study; Figure S2: (A) AMMI 2 biplots of PC1, PC2, and PC3; Figure S3: Calculated values of weighted average of stability (WAASB) and mean performance (Y) (WAASBY); Figure S4: (A) Heatmap of genotypes × environmental interactions, (B) correlation between stability indices; Figure S5: (A) Comparative analysis of JG14 (G42) and ILC1932 (G39) across diverse environments (SVP = 3), (B) genotype plus genotype × environment (GGE) biplot “environment × genotype relationship” view; Figure S6: (A) Genotype ranking according to performance in a specific environment (E10), (B) ranking trial environments based on the relative performance of genotypes G1 and G2; Figure S7: Field pictures of the heat tolerant testing; Figure S8: Graph of monthly average temperature of chickpea growing season of 2021 to 2023. Table S1: List of selected genotypes used in this study with their heat susceptibility index values of individual environment; Table S2: Genotype rankings in respective tested environments; Table S3: Annichiarico environmental index; Table S4: Descriptive statistics, variance components, and genetic parameters for grain yield of 42 genotypes evaluated across the year and locations; Table S5: Genotype code and their yield across environments with their IPCA scores; Table S6: Genotypes mean yield with different stability indices for ranking genotypes under multi-environmental condition; Table S7: Environmental description of the experimental sites; Table S8: Mean monthly temperature and precipitation data of all the locations; Table S9: Mean ± standard deviation of yield traits of individual locations and seasons.

## References

[1] Merga B., Haji J. (2019). Economic Importance of Chickpea: Production, Value and World Trade. Cogent Food Agric..

[2] Salaria S., Bindra S., Singh I., Rani U., Kumar A.S., Gill B.S., Singh S. (2023). Introgression of Morphological, Phenological, and Productivity Traits Along with Disease Resistance from *Cicer pinnatifidum* into Cultivated Chickpea: A Success Story. Euphytica.

[3] Nabati J., Mirmiran S.M., Yousefi A., Zare Mehrjerdi M., Ahmadi-lahijani M.J., Nezami A. (2023). Identification of diverse agronomic traits in chickpea (*Cicer arietinum* L.) germplasm lines to use in crop improvement. Legume Sci..

[4] Krishnamurthy L., Gaur P.M., Basu P.S., Chaturvedi S.K., Tripathi S., Vadez V., Rathore A., Varshney R.K., Gowda C.L.L. (2011). Large genetic variation for heat tolerance in the reference collection of chickpea (*Cicer arietinum* L.) germplasm. Plant Genet. Resour..

[5] Mohammed A., Tana T., Singh P., Korecha D., Molla A. (2017). Management options for rainfed chickpea (*Cicer arietinum* L.) in northeast Ethiopia under climate change condition. Clim. Risk Manag..

[6] Devasirvatham V., Tan D.K. (2018). Impact of High Temperature and Drought Stresses on Chickpea Production. J. Agron..

[7] Pattison A.L., Uddin M.N., Trethowan R.M. (2021). Use of in-situ field chambers to quantify the influence of heat stress in chickpea (*Cicer arientinum*). Field Crops Res..

[8] Osorio E., Davis A.R., Warkentin T.D., Bueckert R.A. (2023). Ovule abortion and seed set of field pea (*Pisum sativum* L.) grown under high temperature. Can. J. Plant Sci..

[9] Gaur P.M., Jukanti A.K., Varshney R.K. (2012). Impact of Genomic Technologies on Chickpea Breeding Strategies. J. Agron..

[10] Gaur P.M., Jukanti A.K., Samineni S., Chaturvedi S.K., Basu P.S., Babbar A., Jayalakshmi V., Nayyar H., Devasirvatham V., Mallikarjuna N. (2013). Climate Change and Heat Stress Tolerance in Chickpea. Climate Change and Plant Abiotic Stress Tolerance.

[11] Mohanty J., Thakro V., Parida S.K., Nair H., Dixit G.P., Jha U.C. (2023). Delineation of Genes for a Major QTL Governing Heat Stress Tolerance in Chickpea.

[12] Singh N.T., Dhaliwal G.S. (1972). Effect of Soil Temperature on Seedling Emergence in Different Crops. Plant Soil..

[13] Kaushal N., Gupta K., Bhandhari K., Kumar S., Thakur P., Nayyar H. (2011). Proline Induces Heat Tolerance in Chickpea (*Cicer arietinum* L.) Plants. Physiol. Mol. Biol. Plants..

[14] Upadhyaya H.D., Dronavalli N., Gowda C.L.L., Singh S. (2011). Identification and Evaluation of Chickpea Germplasm for Tolerance to Heat Stress. Crop Sci..

[15] Devasirvatham V., Gaur P.M., Mallikarjuna N., Raju T.N., Trethowan R.M., Tan D.K. (2013). Reproductive Biology of Chickpea Response to Heat Stress in the Field Is Associated with the Performance in Controlled Environments. Field Crop. Res..

[16] Kumar S., Thakur P., Kaushal N., Malik J.A., Gaur P., Nayyar H. (2013). Effect of Varying High Temperatures during Reproductive Growth on Reproductive Function, Oxidative Stress and Seed Yield in Chickpea Genotypes Differing in Heat Sensitivity. Arch. Agron. Soil Sci..

[17] Chandel S.S., Sharma K.D. (2023). Down-Regulation of Carbohydrate Metabolic Pathway Genes Lowers Sucrose and Starch Content in Chickpea Leaves Under High Temperature Stress. Natl. Acad. Sci. Lett..

[18] Devasirvatham V., Gaur P.M., Raju T.N., Trethowan R.M., Tan D.K.Y. (2015). Field response of chickpea (*Cicer arietinum* L.) to high temperature. Field Crops Res..

[19] Rani A., Devi P., Jha U.C., Sharma K.D., Siddique K.H., Nayyar H. (2020). Developing Climate-Resilient Chickpea Involving Physiological and Molecular Approaches with a Focus on Temperature and Drought Stresses. Front. Plant Sci..

[20] Jeffrey C. (2023). Investigation into Physiology and Genetic Infrastructure Regarding the Impact of Heat Stress on Commercially Cultivated Chickpea. Doctoral Dissertation.

[21] Singh M., Bhardwaj C., Singh S., Panatu S., Chaturvedi S.K., Rana J.C., Rizvi A.H., Kumar N., Sarker A. (2016). Chickpea Genetic Resources and Its Utilization in India: Current Status and Future Prospects. Indian J. Genet. Plant Breed..

[22] Bharadwaj C., Tripathi S., Soren K.R., Thudi M., Singh R.K., Sheoran S., Varshney R.K. (2021). Introgression of “QTL-hotspot” region enhances drought tolerance and grain yield in three elite chickpea cultivars. Plant Genome.

[23] Danakumara T., Kumari J., Singh A.K., Sinha S.K., Pradhan A.K., Sharma S., Jha S.K., Bansal R., Kumar S., Jha G.K. (2021). Genetic Dissection of Seedling Root System Architectural Traits in a Diverse Panel of Hexaploid Wheat through Multi-Locus Genome-Wide Association Mapping for Improving Drought Tolerance. Int. J. Mol. Sci..

[24] Coyne C.J., Kumar S., von Wettberg E.J., Marques E., Berger J.D., Redden R.J., Smýkal P. (2020). Potential and limits of exploitation of crop wild relatives for pea, lentil, and chickpea improvement. Legume Sci..

[25] Farshadfar E. (2008). Incorporation of AMMI Stability Value and Grain Yield in a Single Non-Parametric Index (GSI) in Bread Wheat. Pak. J. Biol. Sci..

[26] Farshadfar E., Zali H., Mohammadi R. (2011). Evaluation of Phenotypic Stability in Chickpea Genotypes Using GGE-Biplot. Ann. Biol. Res..

[27] Adjabeng-Danquah J., Manu-Aduening J., Gracen V.E., Asante I.K., Offei S.K. (2017). AMMI Stability Analysis and Estimation of Genetic Parameters for Growth and Yield Components in Cassava in the Forest and Guinea Savannah Ecologies of Ghana. Int. J. Agron..

[28] Bocianowski J., Warzecha T., Nowosad K., Bathelt R. (2019). Genotype by Environment Interaction Using AMMI Model and Estimation of Additive and Epistasis Gene Effects for 1000-Kernel Weight in Spring Barley (*Hordeum vulgare* L.). Appl. Genet..

[29] Woldemeskel T.A., Fenta B.A., Mekonnen G.A., Endalamaw H.Z., Alemu A.F. (2021). Multi-Environment Trials Data Analysis: An Efficient Biplot Analysis Approach.

[30] Singh C., Gupta A., Gupta V., Kumar P., Sendhil R., Tyagi B.S., Singh G., Chatrath R., Singh G. (2019). Genotype × Environment Interaction Analysis of Multi-Environment Wheat Trials in India Using AMMI and GGE Biplot Models. Crop Breed. Appl. Biotechnol..

[31] Philanim W.S., Kumar A., Shittegar N., Sankar S.M., Bharadwaj C., Ngangkham U., Bhattacharjee B. (2022). Stability Analysis of Yield and Yield Related Traits in Ricebean (*Vigna umbellata* (Thunb.) Ohwi and Ohashi). Indian J. Genet. Plant Breed..

[32] Srivastava A.K., Saxena D.R., Saabale P.R., Raghuvanshi K.S., Anandani V.P., Singh R.K., Dixit G.P. (2021). Delineation of genotype-by-environment interactions for identification and validation of resistant genotypes in chickpea to fusarium wilt using GGE biplot. Crop Prot..

[33] Karkaj F.A., Hervan E.M., Roustaii M., Bıhamta M., Mohammadi S. (2023). Comprehensive Stability Analysis of Wheat Genotypes through Multi-Environmental Trials. J. Agric. Sci..

[34] Olivoto T., Lucio A.D., da Silva J.A., Marchioro V.S., de Souza V.Q., Jost E. (2019). Mean Performance and Stability in Multi-Environment Trials I: Combining Features of AMMI and BLUP Techniques. J. Agron..

[35] Nataraj V., Bhartiya A., Singh C.P., Devi H.N., Deshmukh M.P., Verghese P., Gupta S. (2021). WAASB-based stability analysis and simultaneous selection for grain yield and early maturity in soybean. Agron. J..

[36] Li Z., Wu W. (2023). Genotype recommendations for high performance and stability based on multiple traits selection across a multi-environment in rapeseed. Eur. J. Agron..

[37] Fischer R.A., Maurer R. (1978). Drought Resistance in Spring Wheat Cultivars. I. Grain Yield Responses. Aust. J. Agric. Res..

[38] Neisse A.C., Kirch J.L., Hongyu K. (2018). AMMI and GGE Biplot for Genotype x Environment Interaction: A Medoid-Based Hierarchical Cluster Analysis Approach for High-Dimensional Data. Biom. J..

[39] Harish D., Bharadwaj C., Kumar T., Patil B.S., Pal M., Hegde V.S., Sarker A. (2020). Identification of Stable Drought-Tolerant Landraces of Chickpea (*Cicer arietinum*) Under Multiple Environments. Indian J. Agric. Sci..

[40] Matongera N., Ndhlela T., van Biljon A., Labuschagne M. (2023). Genotype × Environment Interaction and Yield Stability of Normal and Biofortified Maize Inbred Lines in Stress and Non-Stress Environments. Cogent Food Agric..

[41] Roostaei M., Jafarzadeh J., Roohi E., Nazary H., Rajabi R., Mohammadi R., Khalilzadeh G.R., Seif F., Mirfatah S.M.M., Amiri S.S. (2022). Genotype × Environment Interaction and Stability Analyses of Grain Yield in Rainfed Winter Bread Wheat. Exp. Agric..

[42] Smith P.L. (1978). Sampling Errors of Variance Components in Small Sample Multifacet Generalizability Studies. J. Educ. Stat..

[43] Gauch H.G. (1988). Model Selection and Validation for Yield Trials with Interaction. Int. J. Biom..

[44] Gauch H.G., Zobel R.W. (1988). Predictive and Postdictive Success of Statistical Analyses of Yield Trials. Theor. Appl. Genet..

[45] Gauch H.G., Zobel R.W. (1997). Identifying Mega-Environments and Targeting Genotypes. Crop Sci..

[46] Gauch H.G. (2013). A Simple Protocol for AMMI Analysis of Yield Trials. Crop Sci..

[47] Jorben J., Rao A., Bharadwaj C., Nitesh S.D., Tiwari N., Kumar T., Saxena D.R., Yasin M., Sontakke P.L., Jahagirdar J.E. (2022). Multi-Trait Multi-Environment Analysis for Stability in MABC Lines of Chickpea (*Cicer arietinum*). Indian J. Agric. Sci..

[48] Piepho H.P. (1994). Best Linear Unbiased Prediction (BLUP) for Regional Yield Trials: A Comparison to Additive Main Effects and Multiplicative Interaction (AMMI) Analysis. Theor. Appl. Genet..

[49] Gollob H.F. (1968). A Statistical Model Which Combines Features of Factor Analytic and Analysis of Variance Techniques. Psychometrika.

[50] Yan W., Tinker N.A. (2006). Biplot Analysis of Multi-Environment Trial Data: Principles and Applications. Can. J. Plant Sci..

[51] Santchurn D., Badaloo M.G.H., Koonjah S., Dookun-Saumtally A. (2022). Sugarcane breeding and supporting genetics research in Mauritius. Sugar Tech.

[52] Vaezi B., Pour-Aboughadareh A., Mohammadi R., Mehraban A., Hossein-Pour T., Koohkan E., Ghasemi S., Moradkhani H., Siddique K.H.M. (2019). Integrating Different Stability Models to Investigate Genotype × Environment Interactions and Identify Stable and High-Yielding Barley Genotypes. Euphytica.

[53] Bharadwaj C., Satyavathi C.T., Subramanyam D. (2001). Evaluation of Different Classifactory Analysis Methods in Some Rice (*Oryza sativa*) Collections. Indian J. Agric. Sci..

[54] Bharadwaj C., Patil B.S., Sankar S.M., Shimray P.W., Kumar N., Reddy S.P.P., Singhal T., Hegde V., Parida S.K., Roorkiwal M. (2022). Evaluation and Identification of Stable Chickpea Lines for Yield-Contributing Traits from an Association Mapping Panel. J. Agron..

[55] Spoorthi V., Ramesh S., Sunitha N.C., Vaijayanthi P.V. (2022). Prediction of genotype performance for untested years based on additive main effects and multiplicative interaction and linear mixed models: An illustration using dolichos bean (*Lablab purpureus* (L.) Sweet) multiyear data. Ann. Appl. Biol..

[56] Tara Satyavathi C., Bhat K.V., Bharadwaj C., Tiwari S.P., Chaudhury V.K. (2006). AFLP Analysis of Genetic Diversity in Indian Soybean (*Glycine max* (L.) Merr.) Varieties. Genet. Resour. Crop Evol..

[57] Yan W., Holland J.B. (2010). A Heritability-Adjusted GGE Biplot for Test Environment Evaluation. Euphytica.

[58] Lin C.S., Binns M.R. (2010). Concepts and Methods for Analyzing Regional Trial Data for Cultivar and Location Selection. Plant Breed. Rev..

[59] Bharadwaj C., Jorben J., Rao A., Roorkiwal M., Patil B.S., Ahammed S.K., Varshney R.K. (2022). Development of high yielding Fusarium wilt resistant cultivar by pyramiding of “genes” through marker-assisted backcrossing in chickpea (*Cicer arietinum* L.). Front. Genet..

[60] Lin C.S., Binns M.R. (1988). A Method of Analyzing Cultivar × Location × Year Experiments: A New Stability Parameter. Theor. Appl. Genet..

[61] Cadersa Y., Santchurn D., Soulange J.G., Saumtally S., Parmessur Y. (2022). Genotype-by-Environment Interaction for Marketable Tuber Yield in Advanced Potato Clones Using AMMI and GGE Methods. Afr. Crop Sci. J..

[62] Tsenov N., Gubatov T., Yanchev I. (2023). Effect of Genotype-Environment Interaction on Some Important Quality Parameters of Common Wheat (*Triticum aestivum* L.). J. Agric. Sci. Technol..

[63] Carneiro A.R.T., Sanglard D.A., Azevedo A.M., Souza T.L.P.O.D., Pereira H.S., Melo L.C. (2019). Fuzzy logic in automation for interpretation of adaptability and stability in plant breeding studies. Sci. Agric..

[64] Khan M.M.H., Rafii M.Y., Ramlee S.I., Jusoh M., Al Mamun M. (2021). AMMI and GGE Biplot Analysis for Yield Performance and Stability Assessment of Selected Bambara Groundnut (*Vigna subterranea* L. Verdc.) Genotypes Under the Multi-Environmental Trials (METs). Sci. Rep..

[65] Felipe D.M., Muhammad Y. (2022). Agricolae: Statistical Procedures for Agricultural Research, R Package Version 1.4.0. https://github.com/myaseen208/agricolae.

[66] Olivoto T., Lucio A.D.C. (2020). Metan: An R Package for Multi-Environmental Trial Analysis. Methods Ecol. Evol..

[67] Piepho H.P., Möhring J. (2007). Computing heritability and selection response from unbalanced plant breeding trials. Genetics.

