# Supplementary material for: A Multi-Model Based Stability Analysis Employing Multi-Environmental Trials (METs) Data for Discerning Heat Tolerance in Chickpea (Cicer arietinum L.) Landraces
Source: Plants (Basel). 2023 Oct 26;12(21):3691. doi: 10.3390/plants12213691 (PMC10647285; doi:10.3390/plants12213691)
Supplement: Supplementary file 1 [file plants-12-03691-s001.zip › plants-2602622-supplementary.pdf]

Supplementary figures

Supplementary Fig S1. Plots showing the pictorial representation of Genotype and overall mean performance of studied trait at each environment under study.

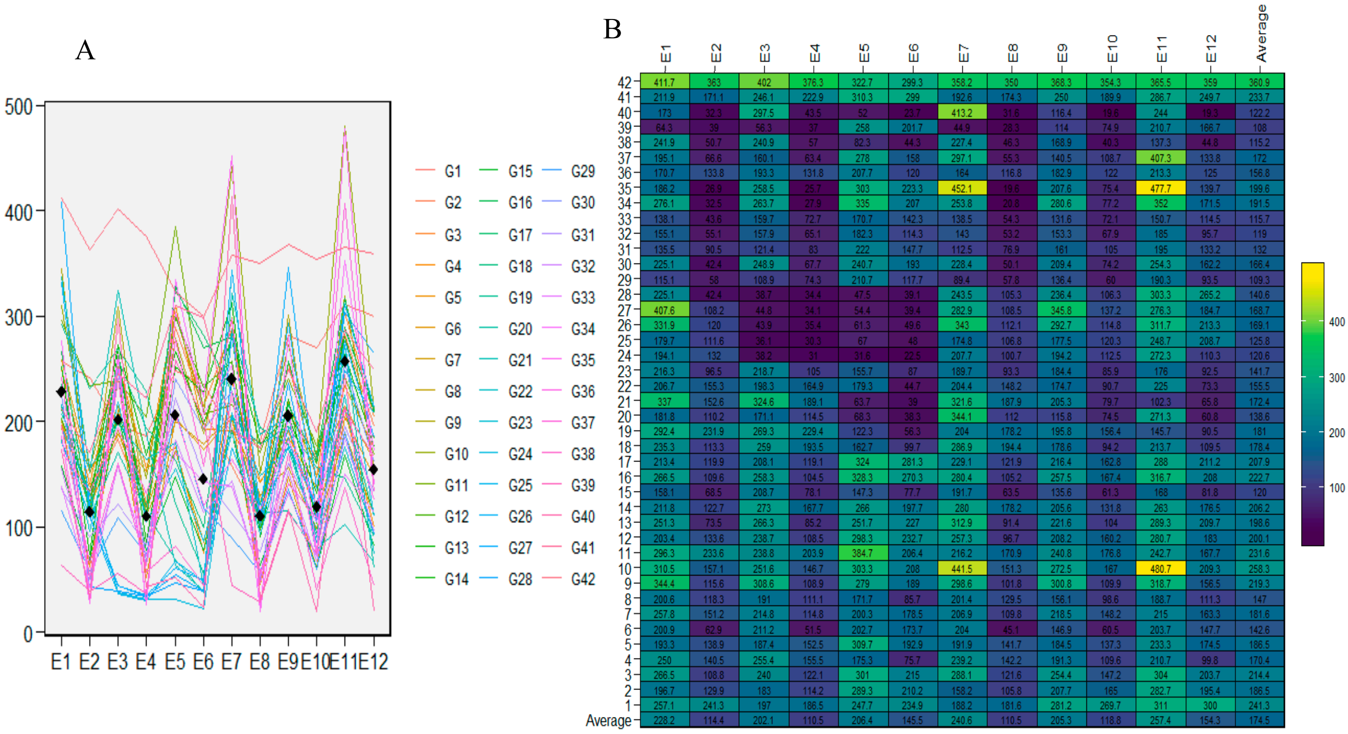

Supplementary Fig S2. (A) AMMI 2 biplots of PC1, PC2 and PC3

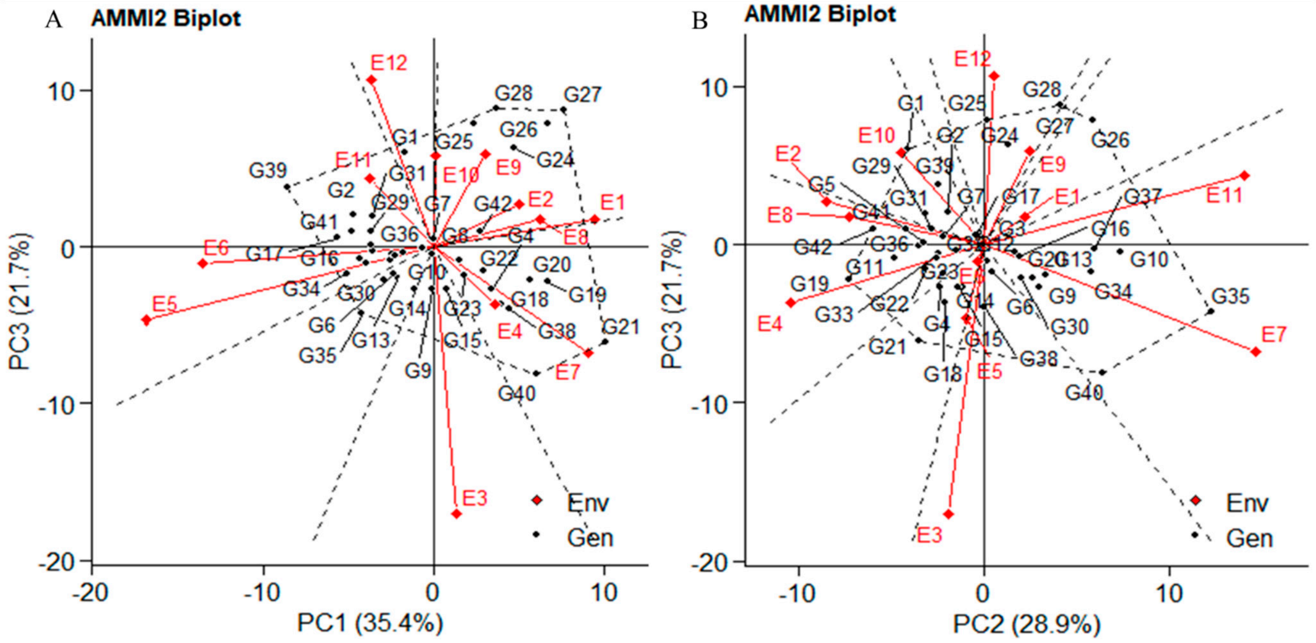

Supplementary Fig S3. (A) displays the calculated values of weighted average of stability (WAASB) and mean performance (Y) (WAASBY) for 42 genotypes, (B) Rankings of 42 genotypes under varying weights for stability and yield. The leftmost ranks reflect stability-based rankings, while the rightmost ranks correspond to grain yield-based rankings. In between

these extremes, the ranks are determined with diverse stability and yield weight combinations. The four clusters represent distinct genotype classes: (1) Genotypes with low productivity and instability; (2) Genotypes with productivity yet instability; (3) Genotypes that are highly productive and stable; and (4) Genotypes that are stable but less productive

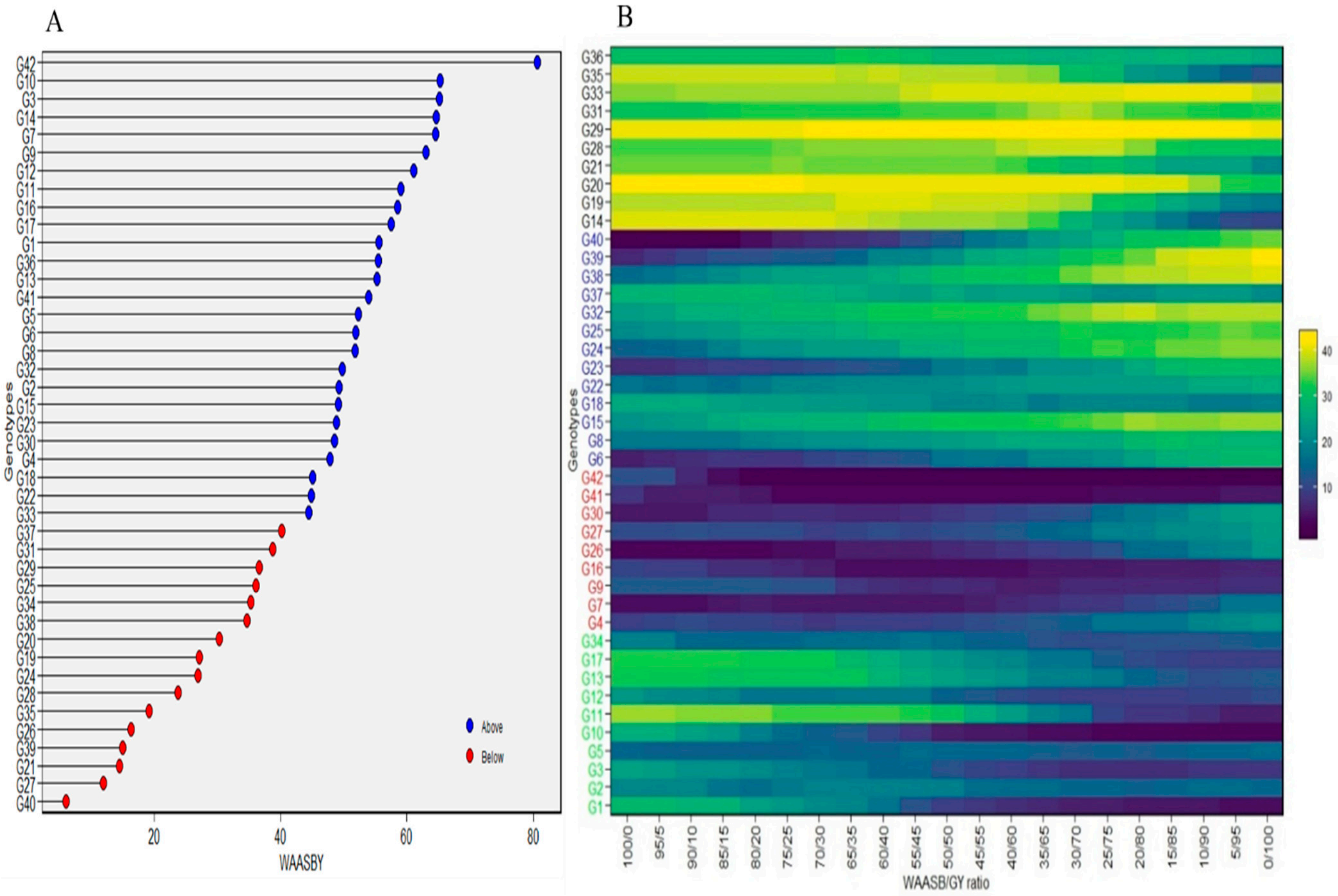

Supplementary Fig S4. (A) Heatmap of Genotypes x environmental interactions, (B) Correlation between stability indices.

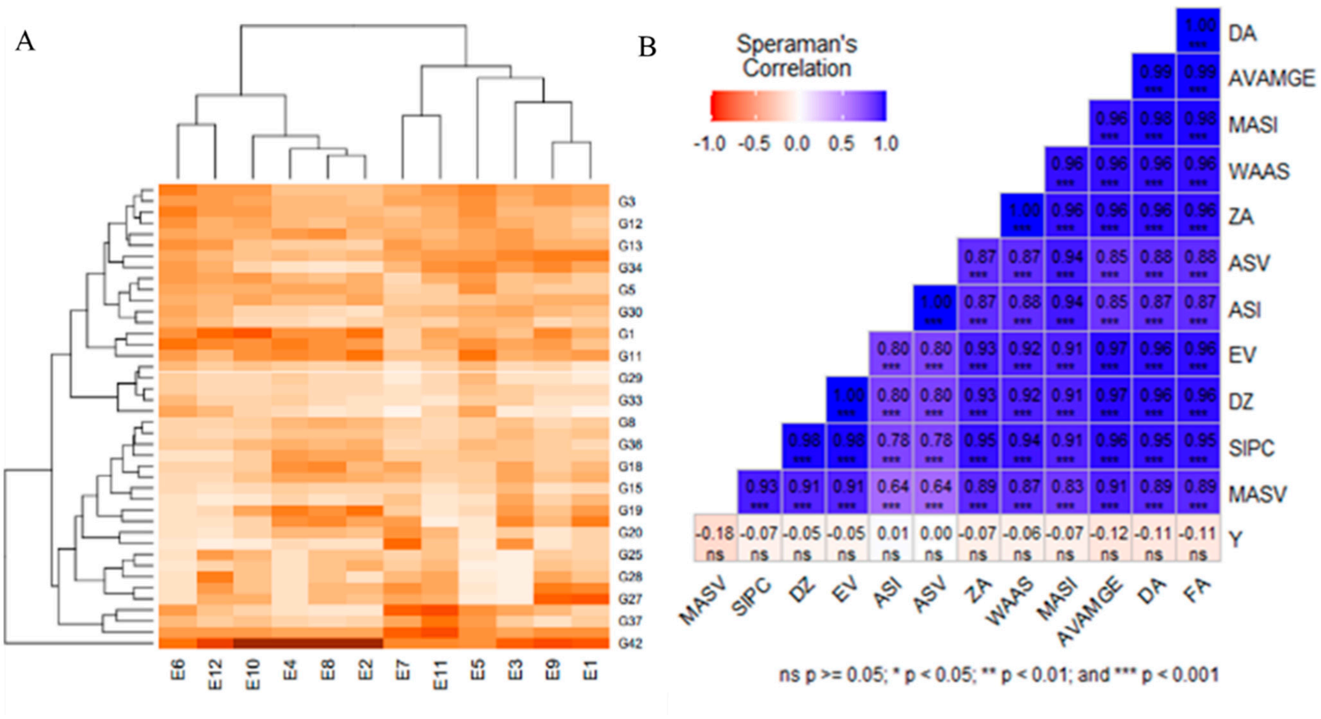

ASI, AMMI Stability Index; ASV, AMMI stability value; AVAMGE, sum across environments of the absolute value of GEI modelled by AMMI; DA, Annicchiarico's D parameter; DZ, Zhang's D parameter; EV, averages of the squared eigenvector values; FA, stability measure based on fitted AMMI model; MASI, Modified AMMI Stability Index; MASV, modified AMMI stability value; SIPC, sums of the absolute value of the IPC scores; ZA, absolute value of the relative contribution of IPCs to the interaction; WAAS, weighted average of absolute scores.

Supplementary Fig S5. (A) Comparative analysis of JG14 (G42) and ILC1932 (G39) across diverse environments (SVP = 3), (B) GGE biplot 'Env. Geno. Relationship' view for 42 genotypes, two seasons, three locations, yield per plot. Biplots: Centering = 2, SVP = 2, Scaling = 0.

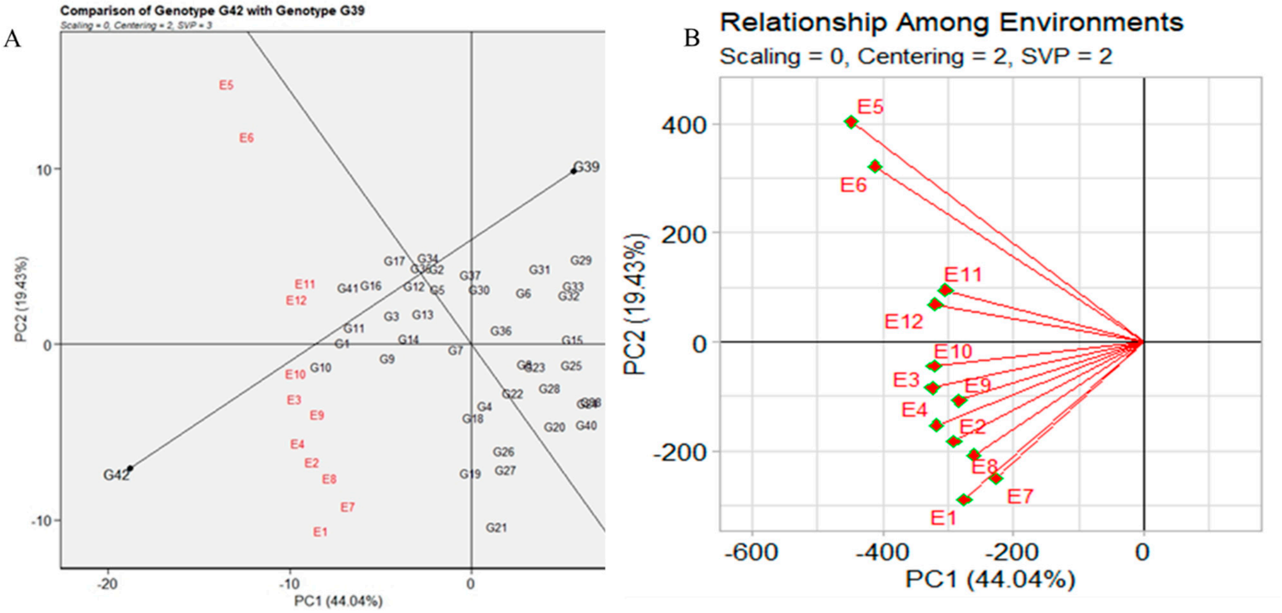

Supplementary Fig S6. (A) Genotype ranking according to performance in a specific environment (E10). (B) Ranking trial environments based on the relative performance of genotypes G1 and G2.

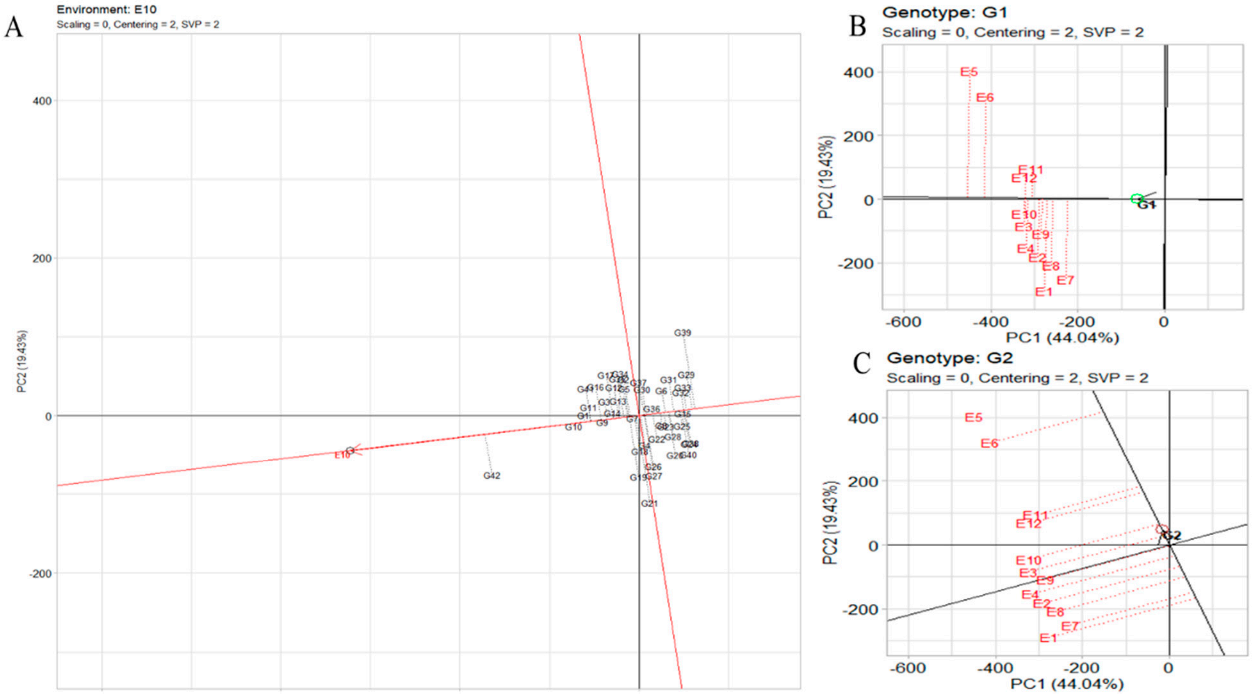

Supplementary Fig S7. Field pictures of the heat tolerant testing

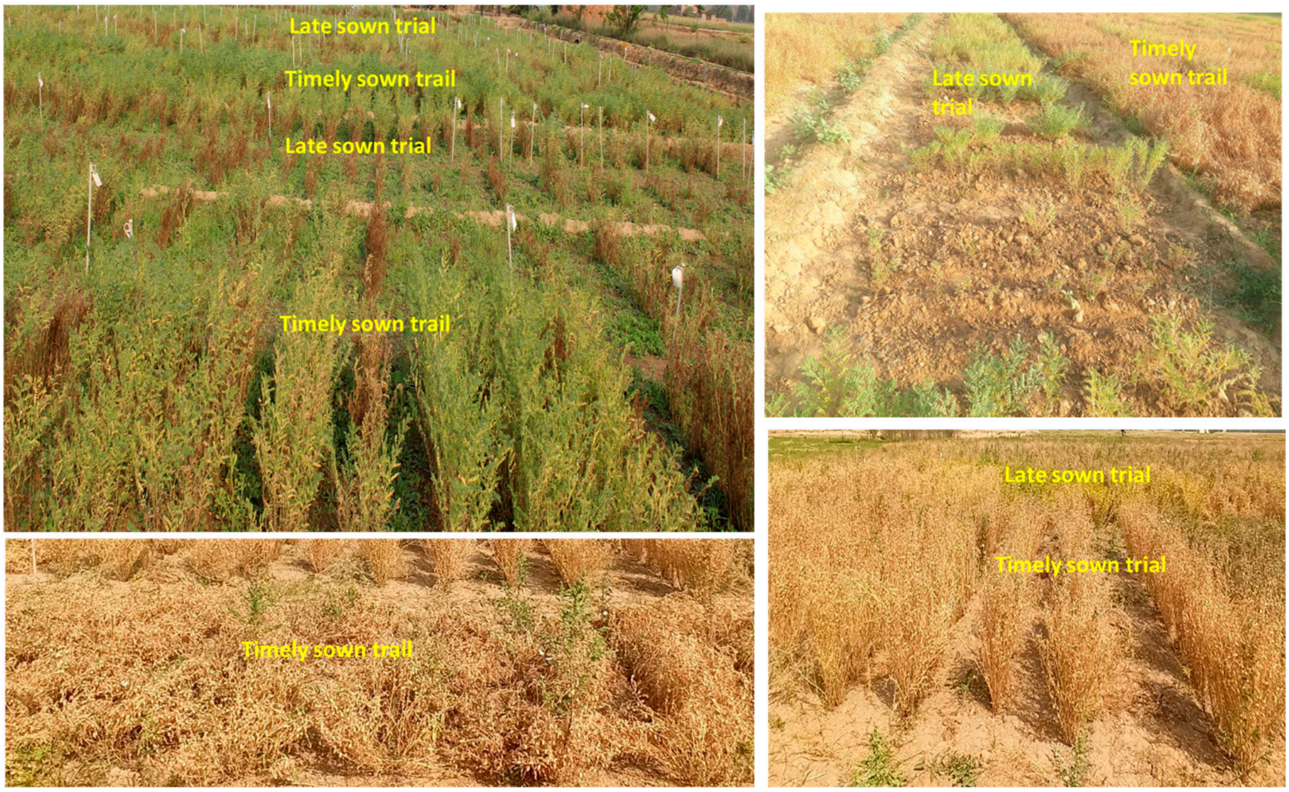

Supplementary Fig S8. Graph of monthly average temperature of chickpea growing season of 2021 to 2023

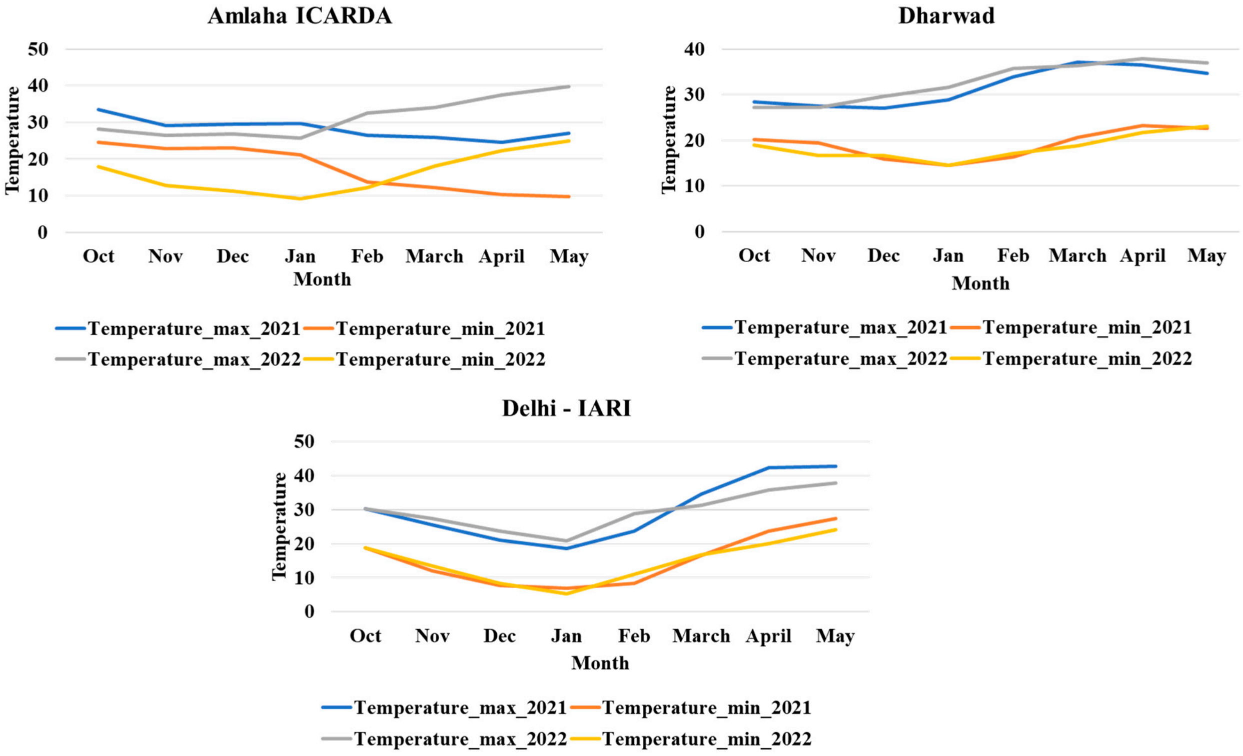

Supplementary tables

Supplementary table S1. List of selected genotypes used in this study with their heat susceptibility index values of individual environment.

S. No.	Accession	Code	Accession Number	Collection/Source	HSI_Amlaha_2021	HSI_Dharwad_2021	HSI_Delhi_2021	HSI_Amlaha_2022	HSI_Dharwad_2022	HSI_Delhi_2022
1	IG5871	G1	5871	Syria	0.12	0.12	0.18	0.07	0.1	0.08
2	IG5842	G2	5842	Jordan	0.68	0.83	0.92	0.62	0.49	0.77
3	IG5852	G3	5852	Jordan	1.19	1.08	0.97	1.07	1	0.83
4	IG5856	G4	5856	Jordan	0.88	0.86	1.93	0.75	1.02	1.32
5	IG5858	G5	5858	Jordan	0.57	0.41	1.28	0.48	0.61	0.63
6	IG5861	G6	5861	Jordan	1.38	1.67	0.49	1.44	1.4	0.69
7	IG5862	G7	5862	Jordan	0.83	1.03	0.37	0.87	0.76	0.6
8	IG5863	G8	5863	Jordan	0.82	0.92	1.7	0.66	0.88	1.02
9	IG5865	G9	5865	Jordan	1.33	1.43	1.09	1.22	1.51	1.27
10	IG5866	G10	5866	Jordan	0.99	0.92	1.06	1.21	0.92	1.41
11	IG5868	G11	5868	Syria	0.43	0.32	1.57	0.39	0.63	0.77
12	IG5874	G12	5874	Syria	0.69	1.2	0.75	1.16	0.55	0.86
13	IG5878	G13	5878	Syria	1.42	1.5	0.32	1.31	1.25	0.69
14	IG5884	G14	5884	Iraq	0.84	0.85	0.88	0.67	0.86	0.82
15	IG5886	G15	5886	Iraq	1.14	1.38	1.61	1.24	1.3	1.29
16	IG5895	G16	5895	Iraq	1.18	1.31	0.6	1.15	0.83	0.85
17	IG5896	G17	5896	Iraq	0.88	0.94	0.45	0.87	0.59	0.67
18	IG5904	G18	5904	Iraq	1.04	0.55	1.32	0.59	1.12	1.22
19	IG5905	G19	5905	Iraq	0.42	0.33	1.83	0.23	0.47	0.95
20	IG5909	G20	5909	Iraq	0.78	0.73	1.49	1.25	0.84	1.94
21	IG5980	G21	5980	Spain	1.1	0.92	1.29	0.77	1.46	0.9
22	IG5993	G22	5993	Greece	0.5	0.38	2.55	0.51	1.14	1.68
23	IG5997	G23	5997	Tunisia	1.11	1.14	1.48	0.94	1.27	1.19
24	IG6000	G24	6000	Tunisia	0.64	0.41	0.98	0.95	1	1.48

25	IG6001	G25	6001	Tunisia	0.76	0.37	0.96	0.72	0.76	0.4
26	IG6002	G26	6002	Tunisia	1.28	0.41	0.67	1.24	1.44	0.79
27	IG6003	G27	6003	Tunisia	1.47	0.5	0.94	1.14	1.43	0.83
28	IG6006	G28	6006	Tunisia	1.63	0.23	0.58	1.05	1.31	0.31
29	ILC0(Austria)	G29		Austria	1.01	0.69	1.5	0.66	1.34	1.27
30	ILC0(Czech)	G30	145219	Czech republic	1.63	1.61	0.66	1.44	1.53	0.9
31	ILC0(Greece)	G31	145218	Greece	0.67	0.69	1.14	0.59	0.81	0.79
32	ILC0(Italy)	G32	145222	Italy	1.29	1.29	1.26	1.16	1.33	1.21
33	ILC0(Lativia)	G33	145228	Latvia	1.38	1.2	0.56	1.12	1.07	0.6
34	ILC0(Russia)	G34	145171	Russian Federation	1.77	1.97	1.29	1.7	1.72	1.28
35	ILC10771	G35	126448	Turkmenistan	1.72	1.99	0.89	1.77	1.51	1.77
36	ILC1312	G36	7150	Cyprus	0.44	0.71	1.42	0.53	0.79	1.03
37	ILC1313	G37	7151	Ethiopia	1.32	1.33	1.46	1.5	0.54	1.68
38	ILC184	G38	6022	Ukraine	1.59	1.69	1.57	1.47	1.81	1.69
39	ILC1932	G39	7770	Jordan	0.78	0.79	0.75	0.67	0.82	0.52
40	ILC239	G40	6077	Ethiopia	1.63	1.88	1.89	1.71	1.98	2.3
41	ILC8666	G41	69656	Portugal	0.38	0.21	0.12	0.17	0.57	0.32
42	JG14	G42	92944	Jabalpur, India	0.23	0.14	0.24	0.04	0.09	0.04

Supplementary table S2. Genotype rankings in respective tested environments

Genotype Ranks	E1	E2	E3	E4	E5	E6	E7	E8	E9	E10	E11	E12
1	G42	G42	G42	G42	G11	G42	G35	G42	G42	G42	G10	G42
2	G27	G1	G21	G19	G34	G41	G10	G18	G27	G1	G35	G1
3	G9	G11	G9	G41	G16	G17	G40	G21	G9	G41	G37	G28
4	G21	G19	G40	G11	G17	G16	G42	G1	G26	G11	G42	G41
5	G26	G41	G14	G18	G42	G1	G20	G14	G1	G16	G34	G26
6	G10	G10	G19	G21	G41	G12	G26	G19	G34	G10	G9	G17

7	G11	G22	G13	G1	G5	G13	G21	G41	G10	G2	G16	G13
8	G19	G21	G34	G14	G10	G35	G13	G11	G16	G17	G26	G10
9	G34	G7	G18	G22	G35	G3	G9	G10	G3	G12	G1	G25
10	G16	G4	G35	G4	G3	G2	G37	G22	G41	G19	G3	G16
11	G3	G5	G16	G5	G12	G10	G3	G4	G11	G7	G28	G3
12	G7	G36	G4	G10	G2	G34	G18	G5	G28	G3	G13	G2
13	G1	G12	G10	G36	G9	G11	G27	G8	G13	G5	G17	G27
14	G13	G24	G30	G3	G37	G39	G16	G17	G7	G27	G41	G12
15	G4	G2	G41	G17	G14	G14	G14	G3	G17	G14	G2	G14
16	G38	G14	G38	G7	G39	G30	G12	G36	G30	G36	G12	G5
17	G18	G26	G3	G20	G13	G5	G34	G26	G12	G25	G27	G34
18	G28	G17	G11	G2	G1	G9	G28	G20	G2	G26	G24	G11
19	G30	G8	G12	G8	G30	G7	G4	G7	G35	G24	G20	G39
20	G23	G9	G23	G9	G31	G6	G17	G27	G14	G9	G14	G7
21	G17	G18	G7	G12	G29	G37	G30	G25	G21	G4	G30	G30
22	G41	G25	G6	G23	G36	G31	G38	G2	G19	G37	G25	G9
23	G14	G20	G15	G16	G6	G33	G11	G28	G24	G28	G40	G6
24	G22	G16	G17	G13	G7	G36	G24	G16	G4	G31	G11	G35
25	G12	G3	G22	G31	G32	G29	G7	G9	G5	G13	G5	G37
26	G6	G27	G1	G15	G22	G32	G22	G24	G23	G8	G22	G31
27	G8	G23	G36	G29	G4	G18	G6	G12	G36	G18	G7	G36
28	G2	G31	G8	G33	G8	G23	G19	G23	G18	G22	G18	G33
29	G37	G13	G5	G30	G33	G8	G8	G13	G25	G23	G36	G8
30	G24	G15	G2	G32	G18	G15	G41	G31	G22	G21	G4	G24
31	G5	G37	G20	G37	G23	G4	G5	G15	G38	G34	G39	G18
32	G35	G6	G37	G38	G15	G19	G15	G29	G31	G35	G6	G4
33	G20	G29	G33	G6	G19	G26	G23	G37	G8	G39	G31	G32
34	G25	G32	G32	G40	G38	G25	G1	G33	G32	G20	G29	G29
35	G40	G38	G31	G39	G20	G22	G25	G32	G6	G30	G8	G23
36	G36	G33	G29	G26	G25	G38	G36	G30	G37	G33	G32	G19
37	G15	G28	G39	G28	G21	G27	G2	G38	G29	G32	G23	G15
38	G32	G30	G27	G27	G26	G28	G32	G6	G15	G15	G15	G22
39	G33	G39	G26	G24	G27	G21	G33	G40	G33	G6	G33	G21
40	G31	G34	G28	G25	G40	G20	G31	G39	G40	G29	G19	G20
41	G29	G40	G24	G34	G28	G40	G29	G34	G20	G38	G38	G38
42	G39	G35	G25	G35	G24	G24	G39	G35	G39	G40	G21	G40

Supplementary table S3. Annichiarico environmental index

Environment	Mean	CV (%)	MSE	H^2	Winning genotype	Annichiarico environmental index	Class
E1	228.2	24.1	3036.8	80.5	G42	53.73	favourable
E2	114.4	26.6	928.0	93.0	G42	-60.11	unfavourable

E3	202.1	31.7	4111.6	80.8	G42	27.62	favourable
E4	110.5	31.7	1224.8	92.0	G42	-64.02	unfavourable
E5	206.4	23.5	2351.3	91.9	G11	31.90	favourable
E6	145.5	32.7	2268.7	89.2	G42	-29.00	unfavourable
E7	240.6	45.5	11991.6	47.8	G35	66.04	favourable
E8	110.5	34.7	1469.6	87.1	G42	-64.01	unfavourable
E9	205.3	31.7	4231.6	61.1	G42	30.77	favourable
E10	118.8	45.0	2858.9	73.4	G42	-55.68	unfavourable
E11	257.4	44.4	13045.8	34.9	G10	82.93	favourable
E12	154.3	39.7	3759.5	73.9	G42	-20.17	unfavourable

Supplementary table S4. Descriptive statistics, variance components, and genetic parameters for grain yield of 42 genotypes evaluated across the year and locations

Maximum	808
Minimum	10.219
Grand Mean	174.5151
Standard Error of Mean (SEm)	56.3282
Critical Difference (CD) 5%	156.2597
Critical Difference (CD) 1%	205.4579
Environmental Variance	9518.592
Genotypic Variance	26454.03
Phenotypic Variance	35972.62
Environmental Coefficient of Variance	55.9054
Genotypic Coefficient of Variance	93.1994
Phenotypic Coefficient of Variance	108.6809
Heritability (Broad Sense)	0.7354
Genetic Advance	287.3247
Genetic Advance as percentage of mean	164.6418

Supplementary table S5. Genotype code and their yield across environments with their IPCA scores

Code	Yield	PC1	PC2	PC3	PC4	PC5	PC6	PC7
G1	241.3	-1.67	-4.13	5.99	-1.08	-1.89	4.60	-0.68
G2	186.5	-4.67	-1.92	2.08	-0.25	0.79	1.59	-0.90
G3	214.4	-2.41	1.64	-0.44	1.74	0.05	-1.59	0.56
G4	170.4	3.39	-2.38	-2.72	-0.15	1.75	-0.09	-1.80
G5	186.5	-3.65	-3.26	0.08	-1.30	1.47	-2.91	1.55
G6	142.6	-2.29	0.39	-1.70	2.18	-2.78	0.45	1.90
G7	181.6	0.01	-2.20	0.51	2.22	-0.73	2.63	2.26
G8	147	1.55	-2.58	-0.85	-1.06	0.57	-0.98	0.19
G9	219.3	-0.07	2.97	-2.69	5.84	2.52	0.82	-2.28
G10	258.3	-0.06	7.34	-0.45	-3.40	3.12	-0.62	0.09
G11	231.6	-2.57	-8.94	-0.87	1.27	6.67	-1.41	3.44
G12	200.1	-3.90	0.18	-1.01	-0.39	-0.70	2.04	2.52

G13	198.6	-2.11	3.31	-1.95	2.19	-3.96	-1.06	1.25
G14	206.2	-1.12	-1.17	-2.65	-1.72	-1.88	-3.23	-0.38
G15	120	0.75	-1.38	-2.66	-0.28	-1.07	0.76	-0.88
G16	222.7	-4.36	1.93	-0.73	2.59	-0.59	0.68	1.85
G17	207.9	-5.59	-0.45	0.56	0.18	-1.20	-0.67	3.05
G18	178.4	3.96	-2.08	-3.65	-2.25	-0.88	-4.85	-1.20
G19	181	6.70	-7.25	-2.21	-0.01	2.21	2.80	-0.13
G20	138.6	5.65	2.58	-2.12	-6.83	0.07	-0.01	1.90
G21	172.4	10.13	-3.51	-6.05	3.07	-1.67	-2.11	1.84
G22	155.5	2.97	-3.15	-1.49	-2.84	4.63	-1.14	-2.83
G23	141.7	1.78	-2.17	-1.80	1.60	0.29	0.66	-1.33
G24	120.6	4.76	1.34	6.30	-3.32	1.81	3.18	0.17
G25	125.8	2.41	0.19	7.82	-2.58	-2.09	0.67	-0.59
G26	169.1	6.71	5.81	7.85	1.57	0.43	-1.77	2.86
G27	168.7	7.68	4.27	8.73	6.69	2.80	-0.78	1.66
G28	140.6	3.65	4.14	8.79	-0.75	-4.43	-3.16	-4.00
G29	109.3	-3.64	-2.82	1.02	-0.95	1.77	-1.27	-1.52
G30	166.4	-2.91	1.97	-2.11	3.66	-2.21	-0.08	-1.47
G31	132	-3.57	-3.15	1.92	-0.68	0.54	-0.06	0.18
G32	119	-1.73	-1.51	-0.38	0.67	0.11	0.11	-0.81
G33	115.7	-2.27	-2.45	-0.58	0.54	-2.81	-0.49	0.90
G34	191.5	-5.05	5.81	-1.70	6.38	2.41	0.69	-4.16
G35	199.6	-4.19	12.28	-4.27	-3.53	0.45	1.10	0.13
G36	156.8	-0.68	-3.47	-0.09	-1.45	1.06	0.90	-1.75
G37	172	-3.54	5.94	-0.22	-4.61	3.78	0.32	-0.04
G38	115.2	4.44	0.00	-3.95	4.02	-1.03	1.68	-0.27
G39	108	-8.57	-2.49	3.83	-1.55	-1.10	-1.04	0.40
G40	122.2	6.05	6.41	-8.10	-3.14	-3.29	2.80	0.23
G41	233.7	-4.77	-4.18	0.99	-0.94	-2.73	-0.76	-0.90
G42	360.9	2.76	-5.98	0.96	-1.34	-2.26	1.59	-1.02
E1	228.2	9.42	2.17	1.80	10.82	4.11	-0.91	2.00
E2	114.4	4.99	-8.45	2.78	-3.93	4.25	5.34	3.18
E3	202.1	1.37	-1.90	-17.04	3.24	-4.13	3.84	-4.43
E4	110.5	3.57	-10.43	-3.69	-4.80	0.61	-3.21	-2.04
E5	206.4	-16.84	-0.97	-4.67	1.43	7.45	-3.45	0.81
E6	145.5	-13.54	-0.35	-1.03	1.96	-5.37	0.88	4.75
E7	240.6	9.08	14.64	-6.73	-4.32	-2.08	-2.20	5.09
E8	110.5	6.23	-7.25	1.77	-4.44	-0.73	-5.98	-0.87
E9	205.3	3.00	2.48	5.90	8.45	1.26	0.07	-3.19
E10	118.8	0.08	-4.45	5.84	-2.40	-0.76	5.28	1.49
E11	257.4	-3.75	14.02	4.38	-7.10	3.83	2.21	-5.01
E12	154.3	-3.61	0.50	10.70	1.08	-8.44	-1.87	-1.77

IPCA, Interaction principal component axis.

Supplementary table S6. Genotypes mean yield with different Stability indices for ranking genotypes under multi-environmental condition

S. No.	GEN	GEN	Y	CV	Shukla	Ecovelence	R^2	ASTAB	ASI	ASV	AVAMGE	DA	DZ	EV	FA	MASI	MASV	SIPC	ZA	WAAS	WAASB	HMGV	RPGV	HMRPGV
1	IG5871	G1	241.34	18.69	3655.56	117674.05	0.09	56.87	1.33	4.61	509.28	187.63	0.31	0.02	35203.89	1.86	19.04	12.86	0.13	3.39	2.54	231.98	1.44	1.38
2	IG5842	G2	186.52	31.19	2032.06	66649.95	0.47	65.61	2.12	7.34	499.74	201.53	0.34	0.03	40614.16	2.14	10.42	11.25	0.10	2.67	2.03	230.02	1.45	1.45
3	IG5852	G3	214.36	34.12	673.32	23946.44	0.91	32.04	1.66	5.75	402.57	147.05	0.22	0.01	21625.08	1.67	7.70	9.51	0.10	2.77	2.22	219.04	1.37	1.32
4	IG5856	G4	170.43	35.35	1639.99	54327.57	0.57	16.42	1.38	4.80	302.90	110.13	0.15	0.01	12128.54	1.40	5.70	5.49	0.06	1.80	1.37	180.99	1.15	1.14
5	IG5858	G5	186.48	25.80	1830.11	60302.94	0.44	23.98	1.21	4.20	377.68	120.98	0.21	0.01	14635.61	1.29	8.08	9.56	0.09	2.45	1.88	168.80	1.11	1.09
6	IG5861	G6	142.55	47.82	949.54	32627.82	0.80	12.63	0.52	1.80	239.24	83.82	0.16	0.01	7025.07	0.79	8.46	6.66	0.06	1.54	1.21	191.86	1.20	1.18
7	IG5862	G7	181.60	24.84	578.04	20951.93	0.79	9.64	0.48	1.66	206.35	76.97	0.13	0.00	5924.96	0.75	8.31	5.07	0.05	1.35	1.03	101.74	0.68	0.66
8	IG5863	G8	146.99	29.01	666.21	23723.14	0.77	29.90	1.64	5.69	393.23	139.21	0.23	0.01	19379.00	1.66	6.84	9.60	0.09	2.62	1.93	197.19	1.26	1.25
9	IG5865	G9	219.31	44.25	2232.36	72944.96	0.92	31.80	1.98	6.88	428.44	154.45	0.21	0.01	23856.30	1.99	7.10	6.78	0.08	2.41	1.80	189.65	1.20	1.18
10	IG5866	G10	258.29	42.95	4029.01	129411.24	0.88	38.37	1.52	5.29	490.62	155.86	0.25	0.02	24293.42	1.73	12.56	11.94	0.12	3.17	2.37	162.37	1.04	1.00
11	IG5868	G11	231.57	26.14	3213.49	103780.43	0.28	102.33	3.16	10.97	821.08	269.00	0.38	0.04	72359.46	3.20	14.32	16.16	0.19	5.31	3.89	166.47	1.09	1.00
12	IG5874	G12	200.11	32.81	1253.60	42183.91	0.70	29.86	1.74	6.05	383.93	146.54	0.20	0.01	21475.37	1.80	8.88	8.92	0.10	2.87	2.10	171.87	1.08	1.07
13	IG5878	G13	198.65	43.44	1708.18	56470.78	0.85	89.78	2.14	7.41	616.42	216.90	0.44	0.05	47045.11	2.24	12.15	17.19	0.15	3.97	2.87	115.65	0.79	0.73
14	IG5884	G14	206.17	26.34	838.64	29142.21	0.73	160.94	3.73	12.93	1039.47	332.20	0.49	0.06	110359.70	3.96	22.80	22.75	0.24	6.58	4.79	144.96	1.01	0.89
15	IG5886	G15	120.03	44.97	542.10	19822.59	0.81	29.02	1.39	4.82	408.18	131.99	0.23	0.01	17421.75	1.44	7.68	10.45	0.10	2.67	2.08	139.27	0.91	0.87

16	IG5895	G16	222.73	36.78	1850.52	60944.27	0.77	13.70	0.89	3.09	264.02	91.50	0.16	0.01	8371.93	0.98	6.72	7.35	0.07	1.89	1.38	126.23	0.81	0.80
17	IG5896	G17	207.94	32.71	2365.10	77116.83	0.50	75.16	1.73	5.99	623.89	213.03	0.36	0.03	45382.30	2.22	20.27	15.72	0.15	3.94	2.94	95.11	0.69	0.61
18	IG5904	G18	178.38	36.15	2624.99	85284.78	0.42	73.66	0.86	2.97	584.51	206.61	0.36	0.03	42689.17	1.91	23.94	13.01	0.11	3.00	2.24	103.76	0.73	0.66
19	IG5905	G19	181.01	39.03	7075.23	225149.44	0.02	142.93	2.91	10.08	909.63	306.58	0.47	0.06	93990.72	3.37	26.32	21.94	0.23	6.30	4.57	131.48	0.94	0.84
20	IG5909	G20	138.58	65.47	4478.53	143538.77	0.48	198.15	2.99	10.36	944.66	341.84	0.61	0.09	116851.18	3.57	29.34	27.37	0.25	6.79	4.96	129.11	0.94	0.83
21	IG5980	G21	172.39	62.82	10615.51	336415.32	0.15	108.33	1.76	6.10	766.37	259.61	0.42	0.04	67399.01	2.60	27.50	17.33	0.17	4.77	3.61	105.58	0.79	0.69
22	IG5993	G22	155.46	36.80	2175.95	71171.96	0.43	23.17	1.53	5.29	361.53	127.71	0.18	0.01	16308.84	1.54	6.67	8.44	0.09	2.57	1.92	93.88	0.63	0.61
23	IG5997	G23	141.73	37.60	745.52	26215.53	0.75	11.67	0.97	3.38	238.59	85.47	0.14	0.01	7305.97	0.99	4.27	6.22	0.06	1.66	1.23	192.42	1.22	1.21
24	IG6000	G24	120.59	68.24	4587.22	146954.91	0.35	30.21	1.18	4.08	363.60	127.23	0.25	0.02	16186.48	1.29	8.59	10.65	0.09	2.49	1.86	132.23	0.92	0.88
25	IG6001	G25	125.78	57.34	4202.70	134869.82	0.27	26.84	1.56	5.40	394.57	136.66	0.20	0.01	18676.10	1.61	8.42	9.32	0.10	2.83	2.07	120.16	0.77	0.76
26	IG6002	G26	169.15	71.83	9071.10	287876.92	0.46	5.86	0.75	2.60	162.33	63.51	0.09	0.00	4032.89	0.76	3.21	4.29	0.05	1.27	0.93	99.86	0.67	0.66
27	IG6003	G27	168.66	76.86	11355.32	359666.52	0.39	11.78	1.07	3.71	241.41	90.81	0.13	0.00	8246.61	1.08	4.66	5.84	0.06	1.80	1.38	98.38	0.66	0.64
28	IG6006	G28	140.60	74.78	7167.14	228038.08	0.38	102.83	2.45	8.50	726.74	239.98	0.46	0.05	57592.77	2.52	12.85	18.94	0.17	4.61	3.41	135.93	1.03	0.94
29	ILC0(Austria)	G29	109.33	45.54	1614.00	53510.76	0.50	199.05	3.85	13.31	1071.51	361.36	0.56	0.08	130580.78	3.96	21.72	24.27	0.24	6.67	4.80	137.01	1.06	0.94
30	ILC0(Czech)	G30	166.37	50.30	1602.31	53143.22	0.84	14.59	1.03	3.57	282.28	96.07	0.16	0.01	9228.57	1.04	4.91	5.68	0.05	1.46	1.10	147.55	0.92	0.91
31	ILC0(Greece)	G31	131.97	33.54	1725.38	57011.22	0.44	69.14	2.13	7.36	596.50	201.58	0.36	0.03	40633.46	2.15	10.15	14.32	0.13	3.59	2.69	138.23	0.95	0.91
32	ILC0(Italy)	G32	119.00	41.95	315.73	12708.05	0.87	51.46	1.57	5.46	504.34	171.79	0.32	0.03	29511.48	1.81	13.73	12.41	0.11	2.92	2.16	85.38	0.63	0.57
33	ILC0(Lativia)	G33	115.74	37.64	900.74	31093.93	0.69	96.68	3.12	10.81	733.83	262.52	0.37	0.03	68918.14	3.23	16.07	16.44	0.18	5.04	3.68	81.39	0.62	0.54
34	ILC0(Russia)	G34	191.51	64.03	5764.80	183964.54	0.86	24.60	1.38	4.80	404.49	129.91	0.19	0.01	16876.12	1.50	9.75	8.64	0.10	2.67	1.98	154.83	0.99	0.96

35	ILC10771	G35	199.64	78.11	12434.77	393592.16	0.81	152.99	2.83	9.81	833.98	311.99	0.50	0.06	97336.18	3.34	27.12	23.69	0.23	6.41	4.72	75.36	0.64	0.52
36	ILC1312	G36	156.77	23.06	896.13	30949.13	0.72	42.08	2.08	7.20	488.92	172.80	0.25	0.02	29859.81	2.09	8.66	10.88	0.12	3.41	2.56	223.17	1.39	1.34
37	ILC1313	G37	172.00	62.25	4303.28	138031.09	0.77	46.14	1.99	6.88	503.03	176.62	0.26	0.02	31195.95	2.00	9.22	11.04	0.12	3.23	2.44	349.27	2.17	2.05
38	ILC184	G38	115.18	72.53	2794.81	90621.96	0.62	25.62	1.60	5.54	374.76	133.45	0.20	0.01	17807.68	1.60	6.37	8.29	0.09	2.52	1.89	175.71	1.09	1.08
39	ILC1932	G39	107.97	74.47	6552.17	208710.38	0.13	13.04	0.82	2.84	241.65	85.84	0.16	0.01	7368.16	0.91	6.27	6.56	0.06	1.56	1.22	114.55	0.80	0.76
40	ILC239	G40	122.17	107.89	9589.72	304176.21	0.60	10.05	0.64	2.20	179.34	71.60	0.15	0.01	5126.29	0.67	3.99	4.95	0.04	0.98	0.77	168.89	1.06	1.05
41	ILC8666	G41	233.70	20.51	3130.18	101162.24	0.18	10.91	0.93	3.21	261.72	84.49	0.13	0.00	7138.18	0.95	4.81	6.04	0.06	1.67	1.24	135.47	0.86	0.85
42	JG14	G42	360.86	8.36	3092.72	99984.73	0.07	50.13	0.86	2.98	465.53	147.69	0.35	0.03	21813.54	1.12	10.75	11.57	0.08	2.02	1.55	188.90	1.22	1.21

Y, Yield; CV, Coefficient of Variation; R^2, Pinthus's (1973) coefficients of determination; ASTAB, AMMI-based stability parameter; ASI, AMMI Stability Index; ASV, AMMI stability value; AMGE, sum across environments of genotype × environment interaction (GEI) modelled by AMMI; AVAMGE, sum across environments of the absolute value of GEI modelled by AMMI; DA, Annicchiarico's D parameter; DZ, Zhang's D parameter; EV, averages of the squared eigenvector values; FA, stability measure based on fitted AMMI model; MASI, Modified AMMI Stability Index; MASV, modified AMMI stability value; SIPC, sums of the absolute value of the IPC scores; Za, absolute value of the relative contribution of IPCs to the interaction; WAAS, weighted average of absolute scores; WAASB, weighted average of absolute scores for the best linear unbiased predictions (BLUPs) of the genotype-vs.- environment interaction; HMGV, harmonic mean of genotypic values; RPGV-relative performance of genotypic values; HMRPGV, harmonic mean of relative performance of genotypic values.

Supplementary table S7. Environmental description of the experimental sites.

Location	Code	Latitude	Longitude	Altitude	Year	Date of sowing	Date of harvesting
Amlaha_Timely	E1	23.14711	76.92035	502 m	2021	15-11-2021	17-04-2022
Amlaha_Late	E2	23.14711	76.92035	502 m	2021	25-12-2021	23-04-2022
Dharwad_Timely	E3	15.45102	75.00844	678 m	2021	27.11.2021	08.03.2022
Dharwad_Late	E4	15.45102	75.00844	678 m	2021	27-12-2021	25.03.2022
Delhi_Timely	E5	28.08	77.12	228.61m	2021	27-11-2021	10-04-2022
Delhi_Late	E6	28.08	77.12	228.61m	2021	27-12-2021	28-04-2022
Amlaha_Timely	E7	23.14711	76.92035	502 m	2022	10-11-2022	16-04-2023
Amlaha_Late	E8	23.14711	76.92035	502 m	2022	22-12-2022	23-04-2023
Dharwad_Timely	E9	15.45102	75.00844	678 m	2022	14.12.2022	25.03.2023
Dharwad_Late	E10	15.45102	75.00844	678 m	2022	15-01-20223	17-04-2023
Delhi_Timely	E11	28.08	77.12	228.61m	2022	27-11-2022	23-04-2023
Delhi_Late	E12	28.08	77.12	228.61m	2022	27-12-2022	05-05-2023

Supplementary table S8. Mean monthly temperature and precipitation data of all the locations

	Amlaha			Dharwad			Delhi		
	Temperature			Temperature			Temperature		
2021-22	Max (°C)	Min (°C)	Precipitation (mm/day)	Max (°C)	Min (°C)	Precipitation (mm/day)	Max (°C)	Min (°C)	Precipitation (mm/day)
October	33.49	24.52	5.55	28.38	20.22	3.96	30.34	18.85	2.5
November	29.03	22.85	11.11	27.52	19.43	3.53	25.62	12.11	0
December	29.43	23.12	8.11	27.1	15.88	1.08	21.09	7.7	0.18
January	29.59	21.06	3.02	28.92	14.46	0	18.62	6.87	2.79
February	26.38	13.63	0.01	33.9	16.3	0	23.67	8.37	0.76
March	25.84	12.2	0.1	37.15	20.69	0.34	34.51	16.49	0
April	24.65	10.38	1.03	36.56	23.17	1.74	42.26	23.76	0.02
May	27.09	9.64	0.03	34.69	22.66	3.37	42.74	27.41	1.31
2022-23									
October	28.14	17.83	3.58	27.22	18.89	3.38	30.31	18.85	3.09
November	26.47	12.77	0	27.12	16.66	0.12	27.38	13.41	0
December	26.86	11.34	0.19	29.69	16.6	0.31	23.74	8.33	0
January	25.77	9.15	0.47	31.65	14.5	0	20.83	5.32	1.17

February	32.53	12.25	0	35.72	17.13	0	28.91	11.06	0
March	33.97	18.16	0.56	36.29	18.83	0	31.31	16.77	2.07
April	37.39	22.26	1.31	37.9	21.65	0.66	35.87	20.11	0.49
May	39.77	24.84	0.83	37.02	23.09	1.57	37.9	24.11	2.57

Supplementary table S9. Mean ± Standard deviation of yield traits of individual locations and seasons.

S. No.	GEN	Code	E1	E2	E3	E4	E5	E6	E7	E8	E9	E10	E11	E12
1	IG5871	G1	257.11±62.34	241.25±62.56	208.84±16.12	211.08±16.11	213.75±12	203.5±9.09	209.98±19.94	217.31±19.58	261.89±58.27	288.33±56.35	313.09±15.38	298.76±13
2	IG5842	G2	196.7±69.54	129.94±40.87	206.79±110.03	239.59±119.3	253±96.28	176.83±104.44	184.83±117.95	233.83±122.33	259±79.18	186.83±95.5	142.97±36.3	123.5±30.57
3	IG5852	G3	266.49±76.58	108.81±13.23	164.28±127.99	212.48±122.41	278.33±30.35	204.66±99.91	223.33±116.71	214.66±112.05	279.66±30.35	210±98.83	221.02±106.57	160.65±103.47
4	IG5856	G4	249.98±89.19	140.48±18.5	181.96±36.71	137.41±54.16	152.66±62.06	129.16±54.29	163.83±39.28	126.83±54.48	158±71.43	138.5±68.29	178.69±45.77	152.2±39.41
5	IG5858	G5	193.25±77.75	138.86±42.72	157.33±96.43	184.97±88.79	203.85±60.42	139.52±60.73	162.33±95.31	176.85±95.35	209.52±48.08	149.19±58.06	137.96±48.19	118.13±37.66
6	IG5861	G6	200.88±93.57	62.88±70.35	101.62±76.93	107.39±80.87	146.66±13.65	97.33±75.24	99.33±77.35	103.66±80.4	152.66±6.8	107.33±76.77	109.09±78.12	64.69±74.18
7	IG5862	G7	257.81±43.06	151.21±30.23	186.74±60.42	169.49±41.81	208.66±26.65	183.5±58.44	185.16±58.75	167.16±38.94	213.33±41.52	192.16±65.54	203.38±66.57	161.93±49.22
8	IG5863	G8	200.62±68.47	118.26±18.44	90.55±58.81	94.28±58.14	126±32.07	111±21.07	130.66±20.59	115.33±31.97	134.66±42.14	131.66±40.8	164.2±21.94	146.98±35.03
9	IG5865	G9	344.4±42.53	115.56±70.54	224.24±137.89	177.6±102.35	262.66±66.49	193.16±130.25	218.5±137.12	173.16±103.05	264±74.08	198.5±132.56	238.19±145.08	152.18±134.19
10	IG5866	G10	310.47±98.86	157.09±35.88	193.56±102.63	211.73±105.27	439±294.24	380.66±348.01	399.33±338.05	219±86.75	443±308.03	388.66±357.35	418.03±341.81	198.89±108.49
11	IG5868	G11	296.33±110.46	233.62±115.87	205.15±74.01	232.95±74.15	227.33±83.73	153±99.18	151.66±97	175.33±98.45	205.33±50.54	166.33±32.74	190.65±74.31	201.08±69.02

12	IG5874	G12	203.4±76.94	133.6±41.23	181.49±101.9	229.77±98.24	259.33±47.98	192.33±88.29	199.66±98.65	220.66±107.02	264.33±33.24	201.33±84.94	151.82±67.22	113.09±7.24
13	IG5878	G13	251.34±113.46	73.45±34.34	144.53±126.04	194.82±137.42	274.66±9.07	204±115.31	202.66±114.27	178.66±97.5	251±42.88	190.33±105.16	163.71±109.97	79.87±43.83
14	IG5884	G14	211.76±60.28	122.74±35.39	161.35±102.25	178±102.82	219±44.3	167.16±45.74	190.16±75.75	191.16±75.76	224±37.72	176.16±47.54	164.18±45.66	122.18±35.02
15	IG5886	G15	158.11±61.96	68.48±23.83	142.08±47.55	112.1±46.72	131±43	95.5±43.61	121.83±54.72	100.16±54.42	137±50.38	105.5±56.15	111.71±52.67	69.99±26.49
16	IG5895	G16	266.54±78.77	109.63±19.21	194.5±120.89	238.26±138.11	303±26.05	228.66±116.03	228.66±116.03	241.66±125.3	304.33±16.86	234±112.52	190.16±90.38	126.17±47.41
17	IG5896	G17	213.43±76.82	119.94±25.16	176.6±119.28	234±127.38	277±53.01	210.16±95.88	208.83±93.95	230.83±103.69	273±31.48	213.16±84.04	162.63±65.49	114.97±18.13
18	IG5904	G18	235.3±105.13	113.31±34.4	134.71±37.8	117.92±39.2	172.33±78.41	154.83±86.93	174.83±73.53	121.5±33.38	177±92.32	163.5±98.99	171.18±91.64	104.26±24.26
19	IG5905	G19	292.36±100.78	231.86±48.65	212.97±94.74	131.16±78.2	103.5±37.61	90.5±37.35	107.83±27.61	82.66±28.29	108.66±49.64	117.83±53.25	163.18±27	170.97±27.26
20	IG5909	G20	181.83±63.98	110.16±23.38	86.96±27.83	62.63±26.89	249.66±349.41	264.16±338.14	268.83±333.54	62.83±33.83	253.66±363.26	272.16±349.07	298.95±323.95	103.74±14.58
21	IG5980	G21	337.03±136.16	152.61±76.72	102.33±48.41	62.33±30.73	86.33±70.88	102.83±66.62	109.5±56.52	63.83±39.67	92±87.42	112.5±81.04	163.5±41.81	133.16±43.76
22	IG5993	G22	206.73±79.61	155.26±69.86	166.25±41.84	134.65±80	152.33±86.07	115±68.43	167±57.26	120.66±81.61	158.66±94.15	125.33±84.05	164.81±53.09	132.68±31.34
23	IG5997	G23	216.25±30.79	96.5±12.41	158.55±58.65	117.91±43.47	135±40.36	104.5±37.41	129.16±46.39	108.83±48.01	142±48.86	115.5±52.39	152.26±59.55	124.97±61.95
24	IG6000	G24	194.08±30.97	132.02±12.57	34.13±7.04	27.36±5.83	117.79±157.03	144.12±141.57	216.33±95.36	148.33±78.11	214.66±109.87	175.33±118.15	201.79±98.4	141.29±29.05
25	IG6001	G25	179.68±27.56	111.55±12.66	35.04±9.85	35.29±9.76	93±92.89	129.66±86.55	187.66±27.64	174.33±28.36	196.66±32.74	182.33±35.27	189.94±30.13	151.48±39.14
26	IG6002	G26	331.91±29.54	120.04±22.99	50.43±9.11	46.47±9.98	153.36±180.01	170.7±168.36	242.33±126.05	167.33±77.88	255±118.5	209±142.31	270.33±134.56	178.67±122.81
27	IG6003	G27	407.62±43.73	108.16±12.75	44.81±7.83	44.56±7.88	116.43±118.37	128.09±110.45	206.66±103.58	194.66±97.43	257±34.69	196±91.32	255.05±150.07	198.59±169.43
28	IG6006	G28	225.05±28.18	42.39±12.6	33.29±5.57	35.68±6.28	110.28±122.93	160.16±109.38	246.41±54.73	247.74±54.99	282.74±23.04	270.15±20.74	251.61±47.28	166.42±114.79

29	ILC0 (Austria)	G29	115.08±39.2	57.96±39.18	120.48±74.09	126.73±72.56	129.66±68.41	70.16±48.88	95.83±90.99	112.83±87.32	133.33±58.24	77.83±43.19	62.68±27.83	47.4±21.25
30	ILC0 (Czech)	G30	225.05±28.18	42.39±12.6	175.94±100.98	148.42±81.48	197.33±29.7	131.5±95.97	149.16±108.94	133.5±99.43	202.33±37.23	140.5±100.67	154.04±107.13	90.17±95.7
31	ILC0 (Greece)	G31	135.5±29.81	90.48±12.64	145.84±98.75	162.97±93.09	173.33±78.48	110.5±45.21	142.16±98.28	159.5±92.33	170.66±76.7	111.83±39.49	101.02±22.6	96.71±23.26
32	ILC0 (Italy)	G32	155.06±30.67	55.13±12.46	132.45±72.74	122.87±71.8	146.33±43.98	92±50.31	117±79.73	113.66±79.5	152.66±39.52	102.33±55.42	110.78±59.85	78.8±52.84
33	ILC0 (Lativia)	G33	138.08±30.63	43.61±12.78	125.36±55.92	127.19±57.13	142.33±31.18	94.5±66.63	97.16±70.26	111.5±77.3	143.33±22.74	99.5±61.86	90.27±51.81	65.43±49.69
34	ILC0 (Russia)	G34	276.09±30.67	32.46±12.55	202.22±165.98	181.6±160.29	257.66±73.81	149.16±122.24	196.5±166.14	177.16±160.97	262.33±73.11	157.83±126.3	182.17±140.92	104.13±135.17
35	ILC10771	G35	186.22±18.12	26.91±13.09	158.15±128.51	162.75±130.2	419.33±321.29	334.33±403.4	357±393.79	159.33±129.24	423.33±335.07	339.66±415.34	340.79±414.75	79.05±103.41
36	ILC1312	G36	170.69±24.44	133.78±12.73	166.32±41.46	147.66±45.34	145.33±47.6	109±16.37	133.33±55.37	135.33±54.37	160±38.74	135±26	161.39±38.71	150.99±41.36
37	ILC1313	G37	195.07±200.54	66.61±12.67	168.8±109.81	183.23±113.94	367.66±210.86	291.83±287.74	309.16±282.68	177.83±116.29	371.66±224.6	299.83±296.01	256.29±323.09	67.74±14.92
38	ILC184	G38	241.89±30.54	50.69±12.38	115.04±99.89	53.2±14.57	108.33±89.85	94.83±100.86	102.16±96	45.16±18.79	113.33±105.3	103.83±112.73	165.97±111.32	104.78±105.53
39	ILC1932	G39	64.25±30.83	39.04±12.97	113.33±128.24	154.62±116.64	157±112.85	76.66±83.85	104.66±132.19	148.66±119.8	160.66±100.75	84.33±74.64	42.93±14.09	35.99±8.43
40	ILC239	G40	172.98±52.49	32.25±9.84	110.53±129.37	28.9±12.44	227.66±347.77	219±355.07	225.66±349.47	19.66±13.31	223.66±347.77	219±351.64	285.13±309.04	88.18±105.17
41	ILC8666	G41	211.92±61.47	171.08±53.94	278.17±39.93	293.65±44.79	257.33±107.68	187.66±113.35	186.33±111.06	218.66±101.47	234.33±77.59	169.66±55.77	186.63±83.94	209.51±81.97
42	JG14	G42	411.66±28.36	363±18.73	330±126.29	248.66±121.7	235.16±98.36	284.83±97.98	350.16±17.06	351.33±17	358.83±8.25	351.5±5.26	376.16±43.47	385.33±40.01
